# Validation of molecular crystal structures from powder diffraction data with dispersion-corrected density functional theory (DFT-D)

**DOI:** 10.1107/S2052520614022902

**Published:** 2014-12-01

**Authors:** Jacco van de Streek, Marcus A. Neumann

**Affiliations:** a Department of Pharmacy, University of Copenhagen, Universitetsparken 2, 2100 Copenhagen, Denmark; b Avant-garde Materials Simulation Deutschland GmbH, Merzhauser Strasse 177, D-79100 Freiburg, Germany

**Keywords:** dispersion-corrected density functional theory, powder data validation, energy mimimization

## Abstract

In 2010 we energy-minimized 225 high-quality single-crystal (SX) structures with dispersion-corrected density functional theory (DFT-D) to establish a quantitative benchmark. For the current paper, 215 organic crystal structures determined from X-ray powder diffraction (XRPD) data and published in an IUCr journal were energy-minimized with DFT-D and compared to the SX benchmark. The on average slightly less accurate atomic coordinates of XRPD structures do lead to systematically higher root mean square Cartesian displacement (RMSCD) values upon energy minimization than for SX structures, but the RMSCD value is still a good indicator for the detection of structures that deserve a closer look. The upper RMSCD limit for a correct structure must be increased from 0.25 Å for SX structures to 0.35 Å for XRPD structures; the grey area must be extended from 0.30 to 0.40 Å. Based on the energy minimizations, three structures are re-refined to give more precise atomic coordinates. For six structures our calculations provide the missing positions for the H atoms, for five structures they provide corrected positions for some H atoms. Seven crystal structures showed a minor error for a non-H atom. For five structures the energy minimizations suggest a higher space-group symmetry. For the 225 SX structures, the only deviations observed upon energy minimization were three minor H-atom related issues. Preferred orientation is the most important cause of problems. A preferred-orientation correction is the only correction where the experimental data are modified to fit the model. We conclude that molecular crystal structures determined from powder diffraction data that are published in IUCr journals are of high quality, with less than 4% containing an error in a non-H atom.

## Abstract 2

The accuracy of 215 experimental organic crystal structures from powder diffraction data is validated against a dispersion-corrected density functional theory method.

## 1. Introduction   

**Figure 1:**
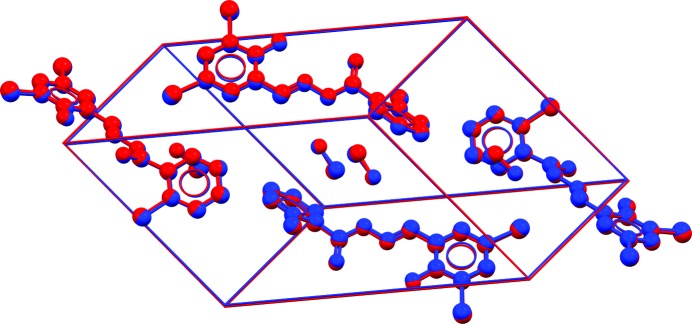
Overlay of a crystal structure with an r.m.s. Cartesian displacement (RMSCD) value of 0.084 Å, illustrating the average reproduction of an experimental single-crystal structure by energy minimization with dispersion-corrected density functional theory with the unit cell free. The H atoms are omitted in the calculation of the RMSCD and in the figure.

In 2010, we published the validation of a dispersion-corrected density functional theory (DFT-D) method for the reproduction of molecular crystal structures against 225 high-quality single-crystal (SX) structures (Van de Streek & Neumann, 2010 ▸). The same computational technique successfully predicted all four target crystal structures in the 2007 blind test of crystal structure prediction (Day *et al.*, 2009 ▸). In the validation study, 225 high-quality crystal structures from SX X-ray data were energy-minimized in full, including the unit-cell parameters. On average, the root mean square (r.m.s.) Cartesian displacement of the non-H atoms upon energy minimization was found to be 0.084 Å (Fig. 1 ▸).

The 2010 validation paper has shown that a DFT-D energy-minimized structure is a good approximation to a SX structure. Therefore, the DFT-D energy-minimized structure of an X-ray powder diffraction (XRPD) structure is a good approximation to what the SX structure would have looked like for that specific XRPD structure. In other words, DFT-D provides us with an approximate SX structure for each XRPD structure! Comparing an XRPD structure with its DFT-D structure therefore allows a semi-quantitative comparison of an XRPD structure with its ‘virtual’ SX structure.

The use of quantum-mechanical calculations to supplement XRPD data is becoming more and more common these days (see *e.g.* Neumann *et al.*, 2002 ▸; Avila *et al.*, 2008 ▸; Florence *et al.*, 2009 ▸; Bekö *et al.*, 2010 ▸; Blanton *et al.*, 2011 ▸, Smrčok, 2012 ▸), but a quantitative benchmark has not been published.

In the current paper, we present the results of DFT-D energy minimizations for 215 molecular crystal structures determined from powder diffraction data and published in one of the IUCr journals. It serves as a quantitative benchmark as to what to expect when combining XRPD and DFT-D, and at the same time provides a thorough quantitative analysis of the quality of molecular crystal structures determined from XRPD data.

## 2. Methods   

The Cambridge Structural Database (CSD, Allen, 2002 ▸) was searched for all organic crystal structures determined from powder diffraction data, both X-ray and neutron, with the atomic coordinates reported, and published in *Acta Crystallographica* *Sections B*, *C* or *E*, the *Journal of Applied Crystallography* or the *Journal of Synchrotron Radiation*. Some new structures were energy-minimized as they were published and the three structures from the publication by Zvirgzdins *et al.* (2014 ▸) have also been included as zb5033_FormA, X and Z (Zvirgzdins *et al.*, 2014 ▸).

A short overview of DFT terminology is helpful here, the reader is referred to *e.g.* the text book by Sholl & Steckel (2009 ▸) and references therein for further information. Of the many flavours of quantum-mechanical (QM) calculations, density functional theory methods are very popular because of their very favourable accuracy *versus* speed trade-off. When a plane-wave basis set is used (as opposed to the more familiar atom-centred basis sets), the electron density is (indirectly) described as a Fourier series, automatically rendering the electron density three-dimensional periodic: an obvious choice for crystals. Within the DFT formalism, different approximations called ‘functionals’, such as ‘PBE’ or ‘BLYP’, have been published. These functionals can be improved on by cleverly mixing in the results from a Hartee–Fock (HF, a non-DFT method) calculation to combine the strengths of the HF and DFT approximations, giving rise to the so-called ‘hybrid functionals’ such as ‘PBE0’ and ‘B3LYP’. It turns out, however, that the HF contribution cannot be evaluated efficiently for plane-wave basis sets and increases computation times by an order of magnitude, and calculations on crystal structures with hybrid functionals are still rare. Last but not least, it needs to be mentioned that the incomplete description of electron correlation in these functionals causes a partial loss of the interactions that keep molecular crystals together: the dispersion or attractive van der Waals interactions; for systems where van der Waals interactions are important, such as molecular crystals, this shortcoming must be corrected for by means of a dispersion correction (Grimme *et al.*, 2010 ▸).

The energy minimizations were carried out with *GRACE* (Neumann, 2013 ▸), *VASP* (Kresse & Furthmüller, 1996*a* ▸,*b* ▸) or *CASTEP* (Clark *et al.*, 2005 ▸). Details of the DFT-D energy minimizations are given elsewhere (Van de Streek & Neumann, 2010 ▸). In brief, the Perdew–Burke–Ernzerhof (PBE) functional (Perdew *et al.*, 1996 ▸) was used with a dispersion correction (Neumann & Perrin, 2005 ▸; Grimme, 2006 ▸; Grimme *et al.*, 2010 ▸). The plane-wave cut-off energy was 520 eV, the *k*-point spacing approximately 0.07 Å^−1^. Inaccurate atomic positions can cause extreme forces in the initial steps of the energy minimization, which can in turn confuse the energy-minimization algorithm. As a result, the minimization may converge to the wrong minimum. To prevent this, the energy minimizations were carried out in stages, gradually releasing more and more degrees of freedom: in the first stage only the intramolecular degrees of freedom were relaxed, in the second stage the unit cell was kept fixed, in the third stage all degrees of freedom including the unit cell were energy minimized simultaneously. *CASTEP* has no option to fix the molecular positions and orientations, so in *CASTEP* a two-stage minimization was used: in the first stage the unit cell was kept fixed, in the second stage all degrees of freedom including the unit cell were energy-minimized simultaneously.

The quantity used for the comparison of the experimental and the energy-minimized crystal structures with the unit-cell parameters included in the energy minimization is the root mean square Cartesian displacement excluding H atoms. ‘Cartesian displacement’ is not uniquely defined when the unit cells of the two crystal structures to be compared are different, as is the case when we compare the experimental crystal structure to the DFT-D optimized structure with the unit cell allowed to vary. In this work the Cartesian displacement for an atom in two crystal structures (1) and (2) is
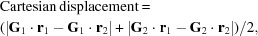
where **r**_*i*_ are the fractional coordinates of the atoms in crystal structure *i*, and **G**_*i*_ is the transformation matrix from fractional to Cartesian coordinates for crystal structure *i*. This definition of Cartesian displacement has the advantages that it is symmetric with respect to the two structures to be compared, that it varies smoothly upon smooth distortions of either or both of the two structures to be compared, and that there is no need for a user-defined parameter such as the number of molecules used for the comparison.

To ensure that the results obtained for the XRPD structures could be compared against those for the SX structures, crystal structures with disorder [BOVDUM (Rohlicek *et al.*, 2009 ▸), CNITBZ02 (Meriles *et al.*, 2000 ▸), FURHUV08 (David, Ibberson *et al.*, 2006 ▸), HEGJAF (Brunelli & Fitch, 2003 ▸), HUSQAN, HUSQIV (Wunschel *et al.*, 2003 ▸), LIWRAK01 (Neumann *et al.*, 2002 ▸), LUKKAD02 (Bhuvanesh *et al.*, 2005 ▸), NIWFEE02 (Derollez *et al.*, 2005 ▸), PAHFIO (Johnston *et al.*, 2004 ▸), SILVAL01 (Helmholdt *et al.*, 2007 ▸), ZZZUXA04 (Damay *et al.*, 2008 ▸), ZZZVCO01, ZZZVCO02 (Damay *et al.*, 2006 ▸) and ZZZURY01 (Jaroń & Grochala, 2011 ▸)] were not attempted, even though experience shows that the main occupancy is always reproduced very well. Although the dispersion correction used in the present paper provides a correction for all elements up to Pu, the elements were restricted to C, H, B, Br, Cl, F, I, N, O and S as in the previous paper. This eliminated ten CSD entries: GUFMOJ, GUFMOJ01 (Si) (Dinnebier *et al.*, 1999 ▸), MEYGII, MEYGII01 (P) (Chernyshev *et al.*, 2013 ▸), PUXLUP01 (P) (Hernandez *et al.*, 2002 ▸), SILVEP (Se) (Helmholdt *et al.*, 2007 ▸), XOZJOM (P) (Chernyshev *et al.*, 2009 ▸), XUDTEV (Tremayne *et al.*, 2002 ▸), ZZZWNG01, ZZZWNG02 and ZZZWNG03 (Si) (Dinnebier *et al.*, 1999 ▸). For most of these structures, the energy-minimized structures can be found in the supporting information, but they are ignored in the analysis part of this paper.

13 reference codes [COTYOA, COTYOA01, COTYOA02, COTYOA03, COTZAN, COTZAN01, COTZAN02, COTZAN03, COTZAN04, COTZAN05, HXACAN26, IBPRAC03 and WEXWUT (Stone *et al.*, 2009 ▸)] correspond to redeterminations of paracetamol and ibuprofen to test a new refinement algorithm and were not energy-minimized.

In the case of discrepancies between the experimental and the energy-minimized crystal structure, no new experimental data was collected for the current paper, but where available the original data was downloaded from the IUCr website. *DASH* (David, Shankland *et al.*, 2006 ▸) was used for crystal-structure solution, *TOPAS* (Coelho, 2012 ▸) was used for Rietveld refinement with restraints and the March–Dollase correction (Dollase, 1986 ▸) was used if deemed necessary. After Rietveld refinement, the positions of the H atoms were energy-minimized with the unit-cell parameters and the positions of the non-H atoms kept fixed. A representative and detailed description of our structure solution and refinement procedure in combination with DFT-D calculations is given in Naelapää *et al.* (2012 ▸). The corrected or re-refined crystal structures were added to the supporting information unless a SX structure is known, in which case the SX structure should be considered the final structure.

The March–Dollase correction requires the user to supply a direction in the form of *h*, *k* and *l*. The directions (100), (010) and (001) are always tried, if only to get a feel for the possible extent of the preferred orientation. Then the BFDH morphology is calculated in *Mercury* (Macrae *et al.*, 2008 ▸) and the directions of the needles or plates, if any, are tried. Finally, the hydrogen-bond network is expanded (again in *Mercury*) to see if this points to any favoured one-dimensional or two-dimensional growth directions, and these directions are then tried. The last two approaches have the advantage of providing a physical explanation for the preferred orientation, especially if the data are flat-plate data. However, none of these three approaches nor their combined use is guaranteed to yield a sensible direction.

If a molecular geometry looked unreasonable, it was checked using *Mogul* (Bruno *et al.*, 2004 ▸) to compare all bond lengths and valence angles against distributions from SX data. The relevant measure is the maximum absolute value of the *z*-score, which measures by how many standard deviations each value in the crystal structure differs from the mean of the distribution from the SX data. Normally, it should be possible to obtain a structure with all values smaller than about 3.

In the course of this work, some inaccuracies in the CSD were discovered. The coordinates of four atoms on special positions in CICYES01 (Ochando *et al.*, 1997 ▸) had been reported as 0.667000 and 0.333000, these should have been 0.666667 and 0.333333. FEFREP, FEFREP02 and FEFREP03 (Halasz *et al.*, 2012 ▸) had been inadvertently marked as containing large voids. GEYREH and GEYREH01 (Pawley & Whitley, 1988 ▸) had been reported in orthogonal coordinates and something had gone wrong during the conversion to fractional coordinates. For HAXMAW and HAXMAW01 (Vella-Zarb & Dinnebier, 2012 ▸), the two-dimensional diagrams incorrectly showed the monomer instead of the dimer. A 2.6 Å C⋯C contact in VANGAT (Brenner *et al.*, 2002 ▸) leads to spurious bonds in *GRACE*; the short contact can be rectified by a minor rotation of the *t*-butyl group. The disordered C atoms that were shown for ZZZVCO03 (Damay *et al.*, 2006 ▸) and ZZZVCO05 (Palatinus & Damay, 2009 ▸) were meant to be deuterium atoms or dummy atoms. WIMWOE (Arnott & Dover, 1968 ▸) had been reported in cylindrical coordinates and something had gone wrong during the conversion to fractional coordinates. Apart from CICYES01 and VANGAT, which correctly reflect the (incorrect) intentions of the authors, these CSD entries have now been edited.

### 2.1. High temperature   

Lattice-energy minimizations lead to static structures and as such refer to *T* = 0 K. For the SX structures, we noticed that all slightly exceptional cases pertained to structures determined at room temperature, whereas structures determined around 120 K were always reproduced very well.

### 2.2. High pressure   

A *PV* term is added to the energy minimization for crystal structures that are the result of experiments conducted at non-ambient pressures.

### 2.3. H atoms   

Due to their low X-ray scattering power, H atoms can hardly be observed directly and their positions are almost invariably the result of some kind of modelling based on assumptions made by the authors. This makes H-atom positions rather uncertain and the quantum-mechanical calculations are ideal for establishing the final H atom locations. Note that the coordinates of the H atoms in the CIF files in the supporting information of this work reflect nuclear positions rather than maxima in the electron density. Three cases that at first glance might appear to be very similar must be clearly distinguished.

#### 2.3.1. Case I: positioning H atoms accurately   

DFT-D calculations are very effective for positioning H atoms (Deringer *et al.*, 2012 ▸), even if the H-atom positions have been reported, even for SX structures. In this work, DFT-D will only be applied if the position of at least one of the H atoms was missing from the original structure.

#### 2.3.2. Case II: resolving ambiguous H-atom positions   

Even if all H atoms are included in the CIF file, occasionally the number and positions of the hydrogen-bond donors and acceptors are such that the hydrogen-bonding network is ambiguous. Although the DFT-D method has never been properly validated for this purpose, both the large energy differences between the alternatives and the large distortions of the non-H atoms in what are presumably the wrong models are usually significant enough for reliable conclusions to be drawn, because the error on the energy differences of polymorphs and the distortions of the non-H atoms have both been documented thoroughly.

#### 2.3.3. Case III: deciding if a structure is a co-crystal or a salt   

Once it has been established which hydrogen bonds are formed, the only ambiguity that remains is whether the proton dissociates to form a salt or whether the structure remains a co-crystal. In our experience, the DFT-D minimizations nearly always clearly favour either the salt or the co-crystal, independent of the initial position of the proton; *i.e.* only one of the two models is a stable local minimum. Unfortunately, comparison with H-atom positions in crystal structures determined from neutron diffraction data shows that the PBE functional is not always reliable and that the decision whether a crystal structure is a co-crystal or a salt cannot be resolved with the DFT-D calculations as presented in this paper. The positions of the non-H atoms are not affected at all and the calculation of the r.m.s. Cartesian displacement with the unit cell free and excluding the H atoms remains a valid measure for the reliability of the crystal structure.

### 2.4. Space-group revisions   

The experimental space group is imposed throughout the energy minimizations; the space-group symmetry can therefore not be lowered as a result of the energy minimization, but additional symmetry elements may be introduced into the structure. All experimental structures (before energy minimization) and all energy-minimized structures were checked for additional symmetry elements with the algorithm in *GRACE*. The search for new symmetry elements was tried with and without taking into account the H or D atoms. The tolerance on all atoms was 0.1 Å. This was also done retrospectively for the SX structures from the 2010 validation study.

## 3. Results and discussion   

### 3.1. Structures that were not attempted or failed   

Some crystal structures could not be energy-optimized.

GLUCIT03 (Rukiah, Lefebvre, Hernandez *et al.*, 2004 ▸): Two —OH groups meet across a twofold axis: no sensible hydrogen-bond pattern is possible within the experimental space group. Even if the space-group symmetry is lowered, a chain of hydrogen bonds remains that both starts and stops at the same OH group and no reasonable hydrogen-bond pattern could be constructed.

HAXJIB (Rácz, 2012 ▸), MUPNEQ, MUPNEQ01 (Pop *et al.*, 2002 ▸): The unit cells contain several hundred Å^3^ of water-accessible voids. None of the dozens of –OH groups and none of the water molecules has H atoms assigned to it. The amount of work that would be needed to assign the positions of the H atoms was considered to be beyond the scope of the current paper.

JEMRUP (van Mechelen *et al.*, 2006*a* ▸), JEMRUP01 (van Mechelen *et al.*, 2006*b* ▸), JEMSAW (van Mechelen *et al.*, 2006*a* ▸), JEMSAW01 (van Mechelen *et al.*, 2006*b* ▸), MEZMOT, MEZMUZ, MEZNAG (Helmholdt *et al.*, 2002 ▸), QESHUS, QESJAA (van Mechelen *et al.*, 2006*a* ▸), QESJAA01 (van Mechelen *et al.*, 2006*b* ▸), QESJEE, QESJEE01, QESJII (van Mechelen *et al.*, 2006*a* ▸), QESJII01 (van Mechelen *et al.*, 2006*b* ▸), QESJOO (van Mechelen *et al.*, 2006*a* ▸), QESJOO01 (van Mechelen *et al.*, 2006*b* ▸), ROLFEE, ROLFEE01, ROLFEE02 (van Mechelen *et al.*, 2008 ▸), XEHQEG (Van Langevelde *et al.*, 2000 ▸). These CSD entries report the crystal structures of triacylglycerols. The extremely soft modes meant that the minimization algorithm was not able to make any progress and after 2 months the energy minimizations were aborted. The unit-cell volumes are well within the range of the DFT-D method and the failure of the optimization is under investigation.

LAQSON (Noguchi *et al.*, 2012 ▸). The positions of the H atoms do not allow for a sensible hydrogen-bonding network in this structure of clarithromycin monohydrate. Meanwhile a correction of the structure based on the original diffraction data has been published (LAQSON01; Van de Streek, 2012 ▸) in which the structure was shown to be a trihydrate, in which the chirality of one of the chiral centres was corrected and in which the positions of the H atoms were adjusted to form a hydrogen-bond network. The LAQSON structure was not considered any further, the RMSCD of the corrected structure LAQSON01 is 0.14 Å.

LIBYAX (Rybakov *et al.*, 2007 ▸). This *Pbca*, *Z*′ = 2 structure has a unit-cell volume of 6251 Å^3^. After 2 months the energy minimization showed little progress and was aborted.

RUJTEW, RUJTIA, RUJTOG (Seijas *et al.*, 2009 ▸). These compounds contain a long *n*-alkyl chain. The first two stages of the energy minimization finish normally, but the final energy minimization with the unit cell free aborts with an error. For some atomic configurations the DFT calculations with the *VASP* program fail for no obvious reason; *GRACE* implements several correction mechanisms to intercept such failures, but on rare occasions there is no appropriate strategy.

### 3.2. RMSCD values: results   

**Figure 2:**
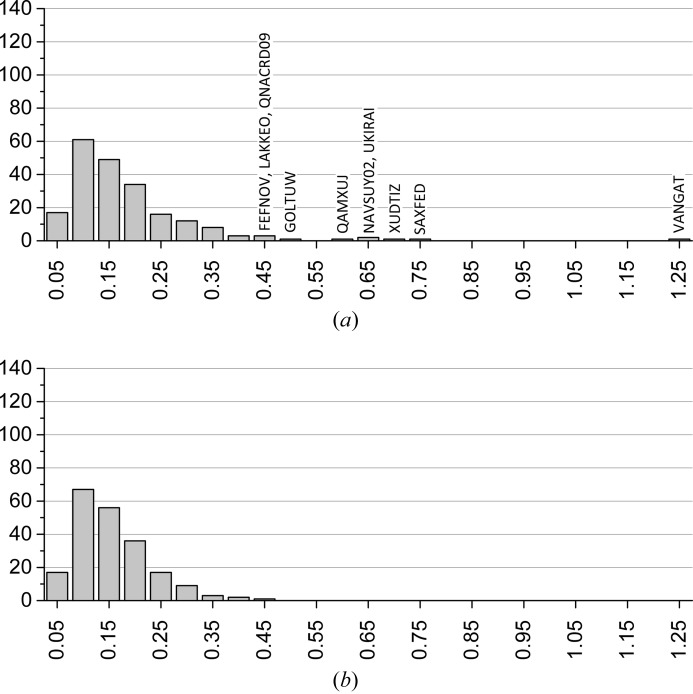
Distribution of RMSCD values for the XRPD structures. (*a*) Original structures. Average: 0.16 Å. (*b*) After correcting or eliminating errors. Average: 0.13 Å.

**Figure 3:**
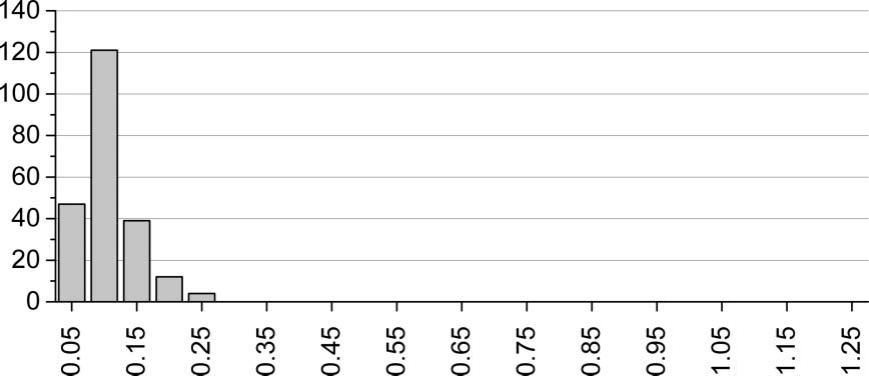
Distribution of RMSCD values for the SX structures after correcting errors. Average: 0.084 Å.

**Figure 4:**
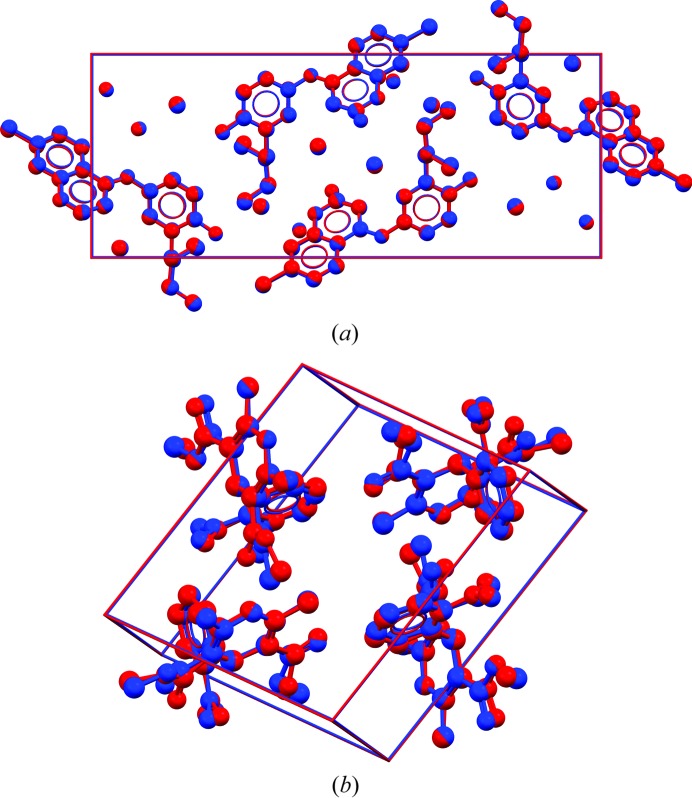
Overlay of representative XRPD and SX structures (by different authors). (*a*) Amodiaquinium dichloride dihydrate, XRPD structure in red [SENJIF (Llinàs *et al.*, 2006 ▸), 298 K, RMSCD = 0.12 Å, *i.e.* the average of the distribution in Fig. 2 ▸], SX structure in blue [SENJIF01 (Mangwala Kimpende & Van Meervelt, 2010 ▸), 100 K]. (*b*) Nifedipine, XRPD structure in red [BICCIZ01 (Bortolotti *et al.*, 2011 ▸), 298 K, RMSCD = 0.34 Å], SX structure in blue [BICCIZ03 (Gunn *et al.*, 2012 ▸), 298 K]. H atoms omitted.

Fig. 2 ▸ presents the distributions of the RMSCD values before and after correcting the errors that could be diagnosed and corrected. The average RMSCD values are 0.16 and 0.13 Å, respectively. The corresponding histogram for the SX structures is given in Fig. 3 ▸. The difference between XRPD and SX structures is further illustrated in Fig. 4 ▸, which shows overlays of two pairs of representative XRPD and SX structures. For the SX structures, RMSCD values below 0.25 Å always indicated a correct structure, while values over 0.30 Å were indicative of structures requiring closer inspection; between 0.25 and 0.30 Å was considered a grey area. Crystal structures with RMSCD values greater than these limits are discussed in detail below.

### 3.3. Structures with 0.25 < RMSCD < 0.30 Å   

FEFREP (0.26 Å)/FEFREP02 (0.25 Å) (Halasz *et al.*, 2012 ▸). The slightly higher r.m.s. values appear to be caused by a minor temperature effect, in combination with considerable preferred orientation for FEFREP02. The structures are correct.

BCHBZP01 (0.26 Å) (Sergeev *et al.*, 2010 ▸). After energy minimization the structure, including the unit cell, is fairly different. The published structure and Rietveld refinement, however, appear to be correct.

IHESUJ (0.26 Å) (Chernyshev *et al.*, 2002 ▸). The unit cell does not change. The diffraction data suffers from severe preferred orientation [March–Dollase parameter *r* > 1.6 in the (010) direction]. The structure is correct.

zb5033_FormX (0.26 Å) (Zvirgzdins *et al.*, 2014 ▸). Re-refinement against the original data yields a structure that is essentially identical with the energy-minimized structure with an r.m.s. value of 0.10 Å. The structure is correct, the re-refined structure can be found in the supporting information.

FOMNAX (0.27 Å) (Papoular *et al.*, 2005 ▸). The geometries of the two molecules in the asymmetric unit changed upon energy minimization. In the published structure, the O atom bends out of the plane of the five-membered ring, whereas in the minimized structure a C atom bends out of the plane. It is mentioned in the original publication that quantum-mechanical calculations on the molecule in vacuum had indeed favoured the geometry consistent with our solid-state calculations and contradicting the published structure. The Rietveld refinement is made more difficult by the 2θ step size, which at 0.029° was slightly too large for the narrow peaks of the high-resolution instrument; as a result, the peak shape appears to be undersampled. Moreover, the diffraction data suffer from severe preferred orientation [March–Dollase parameter *r* > 1.7 in the (011) direction]. Rietveld refinement with *TOPAS* shows that the energy-minimized structure agrees very well with the experimental data, with an r.m.s. of only 0.11 Å. The corrected structure can be found in the supporting information.

WUBDOM (0.28 Å) (Ivashevskaja *et al.*, 2002 ▸). The infinite hydrogen-bonded chains can run in two directions, theoretically introducing an ambiguity. Both models were energy-minimized and both have the same energy and the same positions for the non-H atoms: the H atoms are disordered over two positions. However, this does not explain the slightly higher r.m.s. value of the non-H atoms. Re-refinement against the original data yields a structure that is essentially identical with the energy-minimized structure with an r.m.s. value of 0.08 Å. The original Rietveld refinement might have been hampered by the presence of some preferred orientation [March–Dollase parameter *r* = 0.93 in the (010) direction]. The structure is correct, the re-refined structure can be found in the supporting information.

HUWRUN (0.29 Å) and HUWSAU (0.26 Å) (Lasocha *et al.*, 2010 ▸). The unit cell does not change, the Rietveld refinement was hampered by preferred orientation and it appears that peak asymmetry was not modelled. The crystal structures are correct.

MAQDAJ (0.29 Å) (Kaduk, 2000 ▸). The Rietveld refinement was plagued by peak-shape problems due to stacking faults. The structure is correct.

YEJQAF (0.29 Å) (Chernyshev, Tafeenko *et al.*, 2001 ▸). The unit cell does not change. The diffraction data contains some preferred orientation. The structure is correct.

### 3.4. Structures with RMSCD > 0.30 Å   

WIMWOE (0.32 Å) (Arnott & Dover, 1968 ▸). The structure was obtained by rotating a rigid fragment in the unit cell in discrete steps; no refinement was attempted. The atomic coordinates are not very precise but the structure is correct.

QIKZAN02 (0.32 Å) (Bekö *et al.*, 2014 ▸). The structure is supposed to be very similar to structure QIKZAN01 (Bekö *et al.*, 2014 ▸) from the same paper, but the H atoms form a different network. With the H-atom positions copied from QIKZAN01, QIKZAN02 and QIKZAN01 converge to the same structure with r.m.s. values of 0.26 and 0.13 Å, respectively. Experimentally, a reversible, temperature-dependent phase transition is observed between QIKZAN01 and QIKZAN02, so this is a temperature effect. The structure is correct, but the wrong hydrogen-bonding network was selected.

EPHEDH03 (0.33 Å) (Krebs *et al.*, 2001 ▸). A SX structure is known [EPHEDH02 (Krebs *et al.*, 2001 ▸)] for this crystal structure of ephedrine hemihydrate and it agrees very well with the DFT-D structure with a very low RMSCD value of 0.053 Å. The structure is correct, the high r.m.s. value is caused by a poor Rietveld refinement.

GUFQED (0.33 Å) (Tremayne *et al.*, 1999 ▸). A SX structure is known [GUFQED01 (Clark *et al.*, 2003 ▸)] that proves that the structure is correct. Upon energy minimization, the SX structure shows an r.m.s. value of only 0.16 Å. When the XRPD structure is energy-minimized using *x*,*y*,*z* coordinates the r.m.s. value is only 0.19 Å, confirming that the three-stage energy minimization in delocalized internal coordinates converged to the wrong minimum. The energy of the distorted structure is 2 kJ mol^−1^ lower, which is approximately the error bar of the energy potential used. Surprisingly, GUFQED is not a room-temperature structure but was determined at 120 K.

BICCIZ01 (0.34 Å) (Bortolotti *et al.*, 2011 ▸). A SX structure of this second polymorph of nifedipine is known [BICCIZ03 (Gunn *et al.*, 2012 ▸)], showing that the crystal structure is correct. The published Rietveld refinement looks poor, the difference curve is inconsistent with the observed and calculated profiles suggesting a cut-and-paste error, and the χ^2^ value is 19.5. When the Rietveld refinement is redone with *TOPAS* using the published X-ray synchrotron data, a crystal structure is obtained with an r.m.s. Cartesian displacement of only 0.13 Å. [Note that BICCIZ01 is labelled as ‘polymorph C’ in the CSD whereas BICCIZ03 is labelled as ‘β polymorph’, but the two crystal structures are the same; BICCIZ02 (Gunn *et al.*, 2012 ▸) is the same structure at a different temperature].

DOHREX01 (0.34 Å) (Llinàs *et al.*, 2007 ▸). A SX structure of this second polymorph of sulindac is known [DOHREX03 (Grzesiak & Matzger, 2007 ▸)], which agrees with the XRPD structure. Both energy-minimize to the same structure, but for the SX structure that corresponds to an r.m.s. value of only 0.22 Å. The SX structure was measured at 123 K, which is more similar to the 0 K of the DFT-D method, whereas the XRPD structure was measured at room temperature. There appears to be a minor temperature effect which combined with the slightly less accurate coordinates of the XRPD structure cause a relatively high r.m.s. value for a correct structure.

SUWKIE (0.34 Å) (Kaduk & Golab, 1999 ▸). The structure could not be solved from SX data because of disorder. The powder diffraction data appears to have some preferred orientation.

IBPRAC04 (0.38 Å) (Derollez *et al.*, 2010 ▸). The unit cell does not change. The maximum *Mogul z*-scores for the bond lengths and valence angles are 5.6 and 5.7, respectively, pointing to inaccuracies in the molecular geometry. There is considerable preferred orientation [March–Dollase parameter *r* > 1.2 in the (100) direction] and the isopropyl group appears to be disordered. The structure appears to be correct.

IJEKAJ (0.38 Å) (Ivashevskaja *et al.*, 2003 ▸). With real-space crystal-structure solution in *DASH* we find the same structure. However, Rietveld refinement against the published data reveals considerable preferred orientation [March–Dollase parameter *r* = 0.82 in the (100) direction] and when this preferred orientation is included in the real-space crystal-structure solution, a different structure with a lower χ^2^ (3.9 *versus* 4.3) and more reasonable short contacts is found in three of 50 simulated annealing runs. In the new structure the two C atoms of the central C=C group have rearranged. Rietveld refinement on the new structure reveals severe preferred orientation: the final March–Dollase parameter *r* is 0.69 in the (100) direction. The r.m.s. value of the new structure is 0.14 Å, its energy is 1.4 kJ mol^−1^ more favourable. The corrected structure can be found in the supporting information.

**Figure 5:**
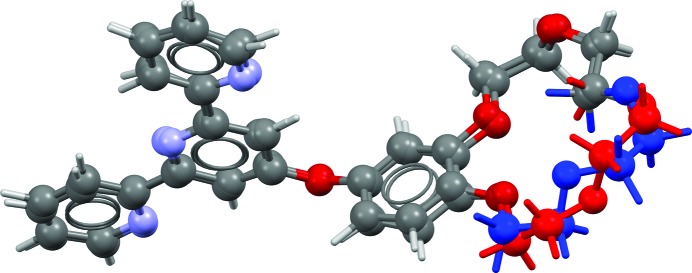
YIRVOL (Logacheva *et al.*, 2008 ▸): overlay of the original (red) and corrected (blue) experimental structures.

YIRVOL (0.39 Å) (Logacheva *et al.*, 2008 ▸). Two C atoms rearranged. Rietveld refinement starting from the energy-minimized structure gave a slightly different structure, the energy minimization of which yielded yet another slightly different structure. This final structure was stable upon Rietveld refinement with an RMSCD value of only 0.14 Å (see Fig. 5 ▸). No preferred orientation correction was necessary. The lattice energy of the corrected structure is more favourable by 25 kJ mol^−1^. The corrected structure can be found in the supporting information.

FEFNOV (0.41 Å) (Bushmarinov *et al.*, 2012 ▸). The unit cell contracts by 3% in the **a** direction, indicating a minor temperature effect. The diffraction data suffer from severe preferred orientation [March–Dollase parameter *r* > 1.4 in the (100) direction]. The structure appears to be correct.

LAKKEO (0.44 Å) (Lefebvre *et al.*, 2005 ▸). A SX structure is known [LAKKEO01 (Guiry *et al.*, 2008 ▸)] that shows that the orientation of one of the C—O groups is off by 180°. Using *DASH*, we find the correct structure. The positions of the H atoms in the SX structure do not form a reasonable hydrogen-bond network and the r.m.s. value of the SX structure is 0.35 Å. With the correct H atom positions, the r.m.s. value is only 0.08 Å, the lattice energy is 13 kJ mol^−1^ lower. The SX structure with the corrected H-atom positions can be found in the supporting information.

QNACRD09 (0.45 Å) (Buchsbaum & Schmidt, 2007 ▸). This is a deliberate error that was published to show that a satisfactory Rietveld refinement is not always proof of a correct structure. The correct crystal structure is known and has an r.m.s. Cartesian displacement of 0.06 Å.

GOLTUW (0.46 Å) (Chernyshev, Yatsenko *et al.*, 1999 ▸). The H atom of the NH group has two possible positions, changing to the alternative position gives a structure with an r.m.s. value of 0.11 Å and a lattice energy that is 15 kJ mol^−1^ lower. The corrected H atom positions are given in the supporting information.

QAMXUY (0.57 Å) (Mora *et al.*, 2005 ▸). The difference curve of the Rietveld refinement in the original publication shows some major discrepancies. The positions of the H atoms are unambiguous. Energy optimization in *x*,*y*,*z* coordinates rather than internal coordinates leads to a distortion of 0.40 Å. The experimental data is not available in the supporting information and the authors did not respond to a request for the data. The crystal structure appears to be incorrect.

NAVSUY02 (0.63 Å) (Noguchi *et al.*, 2012 ▸). The positions of the H atoms are suboptimal and can be improved to give a structure with an RMSCD value of 0.38 Å upon energy minimization that is 16 kJ mol^−1^ lower in energy. A value of 0.38 Å is still high and suggests another H-atom related problem, but the positions of the non-H atoms of the structure appear to be correct.

UKIRAI (0.65 Å) (Hangan *et al.*, 2010 ▸). The crystal structure contains an unsatisfied hydrogen-bond donor and a 2.8 Å N⋯O=S contact. After repositioning the H atom, an excellent Rietveld refinement can be obtained, the new model has an r.m.s. value of only 0.13 Å and the agreement with the experimental ss-NMR data is also much improved (Li *et al.*, 2014 ▸).

XUDTIZ (0.66 Å) (Tremayne *et al.*, 2002 ▸). A SX structure is known [XUDTIZ01 (Zhou *et al.*, 2004 ▸)], which shows that an O atom and an N atom have been swapped. The possible N/O ambiguity is not mentioned in the original paper. Simulated annealing with *DASH* using the published powder diffraction pattern shows that half of the solutions end up with the incorrect molecular geometry. Rietveld refinement on the published data reveals considerable preferred orientation [March–Dollase parameter *r* = 0.86 in the (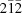) direction, which is the normal to the plane containing the N—H⋯O=S hydrogen bonds]. When this preferred orientation is included in the real-space crystal-structure solution in *DASH*, the correct molecular geometry is found in 50 out of 50 simulated annealing runs. The r.m.s. of the corrected structure, Rietveld refined against the original diffraction data, is 0.17 Å.

SAXFED (0.72 Å) (Burley, 2005 ▸). There is a C/N ambiguity in this crystal structure of glipizide that was mentioned in the original paper, but at the time the author had no instrument available to select the appropriate model. The alternative model, Rietveld refined against the published diffraction data, has an r.m.s. value of 0.13 Å and an energy that is 19 kJ mol^−1^ lower. The corrected structure can be found in the supporting information.

VANGAT (1.21 Å) (Brenner *et al.*, 2002 ▸). As published, the structure contains a 2.6 Å C⋯C contact, the conformation of one of the C(=O)—O—C groups is unusual, the maximum *Mogul z*-score is 6.5, there is some preferred orientation and the publication describes problems with a possible phase transition taking place during the diffraction measurements. We were able to find a structure with a lower *R*_wp_ value, fewer short contacts throughout the structure, an r.m.s. value of 0.67 Å and a lattice energy that is 6 kJ mol^−1^ lower. It therefore seems reasonable to assume that the published structure is incorrect, but we were not able to find a structural model that could reasonably be assumed to be correct.

### 3.5. RMSCD values: discussion   

On the one hand, Fig. 2 ▸ shows that the less accurate atomic coordinates of XRPD structures lead to systematically higher r.m.s. Cartesian displacement values on average. The upper limit on the RMSCD values for a correct crystal structure, which is 0.25 Å for SX structures, must be increased to 0.35 Å for structures from XRPD. The grey area must be extended from 0.30 to 0.40 Å. On the other hand, it is clear from the data that for 95% of all molecular crystal structures determined from powder diffraction data the quality of the structure is comparable to that of a SX structure. It is therefore an interesting question whether some crystal structures determined from XRPD are inherently less well defined than SX structures. For example, when crystals shatter due to the physical treatment necessary for the preparation of a particular polymorph, such as heating, cooling or grinding, the reason behind the need for a powder study may simultaneously explain why the quality of that crystal structure can never equal that of a SX study. Our data suggests that this may be the case for at most 5% of all structures determined from XRPD; for the majority of cases suspicious structures are just the result of substandard data collection, substandard structure solution or substandard Rietveld refinement.

A significant difference between the 225 SX structures from the 2010 validation study and the 215 XRPD structures in the current paper is that the SX structures were all sourced from the August 2008 issue of *Acta Crystallographica Section E*, whereas the XRPD structures were published between 1968 and 2014. In a previous validation study of crystal structures in the Cambridge Structural Database, we showed that the quality of SX structures varied with time (Van de Streek, 2006 ▸). Attempts to discover a similar trend for XRPD structures failed because of an insufficient number of structures. Another difference is that approximately 50% of the SX structures had been determined at low temperature (< 150 K), which is easier to reproduce for the static energy minimizations. For the XRPD structures, only 25% had been determined at low temperature, introducing a slight bias towards slightly higher RMSCD values.

The strength of the DFT-D method is the detection of possible problems; but only on rare occasions – FOMNAX is a good example – is the DFT-D calculation of direct assistance in determining the actual cause of the problem. For the vast majority of cases, it is still up to the experience and imagination of the crystallographer to come up with a list of alternative models that can be tested. For some of the structures presented here, such as IJEKAJ where only 1.5% of all structure solution runs gave a correct solution and only when the structure solution was biased with the correct preferred orientation, finding a better model took expertise, tenacity and several months of hard work. In the same context, it is worth mentioning that energy optimization with DFT-D is very sensitive even for small inaccuracies in a structure; as a result, even a minor error in the position of a H atom can lead to a major distortion in the energy-minimized crystal structure, and there is no relationship between the degree of the distortion and the severity of the error. On the one hand, this feature is a strength, as it makes it virtually impossible even for slight shortcomings in the model to slip through the maze; but at the same time it is a weakness, because it puts insignificant oversights and real errors on equal footing – without giving the user a hint as to which is which. NAVSUY02, for example, appears to be a high-quality crystal structure from synchrotron data supported by an excellent Rietveld refinement, but as long as the correct hydrogen-bonding pattern remains elusive, the structure as a whole will never pass the DFT-D check.

Preferred orientation is the greatest source of problems and uncertainties. A preferred-orientation correction ignores the structural model (the atomic coordinates and the unit cell) and adjusts the calculated structure factors directly so as to fit the experimental data; the one-to-one relationship between the measured intensities and the structural model is lost. This is mathematically equivalent to stating that a preferred-orientation correction modifies the experimental intensities to fit the model – without changing the model. More importantly, severe preferred orientation suppresses the intensities of reflections for particular directions: the information in the powder pattern is not merely redistributed, it is irretrievably lost. Clearly, the presence of preferred orientation should be regarded with much more suspicion than is currently the norm. The authors of papers reporting crystal structures from XRPD that required a substantial preferred-orientation correction during Rietveld refinement should ask themselves: if I am prepared to ignore the experimental data in favour of my model, why did I measure the experimental data in the first place? The use of spherical harmonics for the preferred-orientation correction may obscure the underlying physics, whereas the March–Dollase model retains a link with the physics of the sample. A March–Dollase *r* value between 0.8 and 1.2 appears to be relatively harmless, and, when discovered, must of course be included in the refinement (as the use of a least-squares refinement requires that the model be complete, *i.e.* that the experimental data is described exactly by the model with the exception of the random noise). Values greater or smaller than that should be treated with suspicion; ideally, such data should be discarded, but at the very least the structure solution should be repeated from scratch with the preferred orientation imposed.

One of the 215 crystal structures (GUFQED) converged to the wrong minimum, for the SX structures one of the 225 crystal structures converged to the wrong minimum. There is therefore no difference between XRPD and SX in this respect in spite of the slightly less accurate atomic coordinates of XRPD structures, presumably because of the controlled manner in which the energy minimization proceeds.

### 3.6. High pressure   

Four structures, LSERIN22, LSERIN23, LSERIN24 and LSERIN25 (Moggach *et al.*, 2006 ▸), were measured at pressures of 4.5, 5.2, 7.3 and 8.1 GPa, respectively. Upon energy minimization with the experimental pressure imposed, the r.m.s. values are 0.07, 0.14, 0.28 and 0.23 Å, respectively, *i.e.* it appears that LSERIN24 is not reproduced that well. Minor errors in the DFT-D potential can upset the balance between the DFT-D contribution and the *PV* term, and as a result a structure may be better reproduced at a slightly different pressure. Therefore, LSERIN24 was energy-minimized at a range of pressures, which revealed that the experimental structure is computationally reproduced very well at 4, 5 and 6 GPa (average r.m.s. = 0.16 Å), but changes abruptly at 7 GPa (r.m.s. = 0.27 Å).

The structures are correct, but minor errors in the DFT-D potential may cause the pressure at which a structure is reproduced computationally to differ from the experimental pressure.

### 3.7. H atoms   

#### 3.7.1. Case I: missing H atoms   

GUFQED, HUWSAU, UKIRAI, VANGAT and WIMWOE have been dealt with above. Structures for which the positions of the H atoms are ambiguous are treated below. SX structures are known for BPHENO02 (Kutzke *et al.*, 2000 ▸), CYCHEX07 (Wilding *et al.*, 1993 ▸) and SIKLIH06 (Muangsin *et al.*, 2004 ▸). This leaves CEWVOP01 (Reck *et al.*, 1988 ▸), CNITBZ01 (Meriles *et al.*, 2000 ▸) and WOVTEG (Chernyshev, Paseshnichenko *et al.*, 2001 ▸), for which the H-atom positions after energy minimization with the non-H atoms and the unit cell fixed are given in the supporting information.

#### 3.7.2. Case II: ambiguous H-atom positions   

GOLTUW, LAKKEO, WUBDOM and QIKZAN02 have been dealt with above.

FANDOO (Rukiah, Lefebvre, Descamps *et al.*, 2004 ▸). As reported, the NH_2_ H atoms point towards each other. The corrected H-atom positions are given in the supporting information.

FOGVIG02 (Shankland *et al.*, 2002 ▸). The H atoms of the O_2_S—NH_2_ group do not form hydrogen bonds, which can be resolved by a minor rotation. A SX structure [FOGVIG03 (Florence *et al.*, 2003 ▸)] is known.

LIPVUB/LIPVUB01/LIPVUB02 (Chernyshev, Fitch *et al.*, 1999 ▸). In the paper the crystal structure is determined from laboratory data, from synchrotron data and from neutron data. The three crystal structures are the same, including the positions of the H atoms. However, in spite of the availability of neutron data, the H atoms of the NH_2_ group rearrange upon energy minimization to form a more plausible hydrogen-bond network. After Rietveld refinement with *TOPAS* using the published neutron powder data the same rearrangement of H atoms is observed, confirming that the original refinements had not located the correct H-atom positions for the NH_2_ group. The corrected H-atom positions are given in the supporting information.

MEXZOG (0.16 Å) (Derollez *et al.*, 2013 ▸). The positions of the H atoms as reported constitute an unusual geometry for the CH_2_—O—H group. Two directions are possible for the infinite chain of hydrogen bonds, changing to the alternative direction fixes the awkward CH_2_—O—H geometry, reduces the r.m.s. value upon energy minimization from 0.16 to 0.07 Å and reduces the lattice energy by 5 kJ mol^−1^. The corrected H-atom positions are given in the supporting information.

QIBQIB01 (Tanahashi *et al.*, 2001 ▸). No positions were reported for the H atoms. Two alternatives are possible for the carboxylic acid dimer, and two directions for the infinite chain of hydrogen bonds, for a total of four different models. One model has the lowest energy and the lowest r.m.s. value at 0.09 Å, the least likely model has an r.m.s. value of 0.31 Å and a lattice energy that is 2 kJ mol^−1^ higher. The H-atom positions are given in the supporting information.

XARNAG (Bhuvanesh *et al.*, 2005 ▸). No positions were reported for the H atoms. Two alternatives are possible for the infinite chain of hydrogen bonds. One model has an r.m.s. value of 0.27 Å, the other has an r.m.s. value of 0.21 Å and a lattice energy that is 4 kJ mol^−1^ more favourable. The H-atom positions are given in the supporting information.

#### 3.7.3. Case III: co-crystal or salt   

For two reference code families the proton jumps upon energy minimization: reference codes OXACDH31 (Putkonen *et al.*, 1985 ▸), OXACDH32, OXACDH33 (Lehmann *et al.*, 1994 ▸) and LIPWEM (Chernyshev, Fitch *et al.*, 1999 ▸). All four were determined from neutron diffraction data and the experimental deuterium positions must therefore be assumed to be correct; *i.e.* the calculated deuterium positions are wrong. The PBE functional is known to overbind hydrogen bonds and better functionals, such as the B3LYP hybrid functional, may give better results, but calculations with these functionals are currently too slow when applied with a plane-wave basis set.

**Figure 6:**
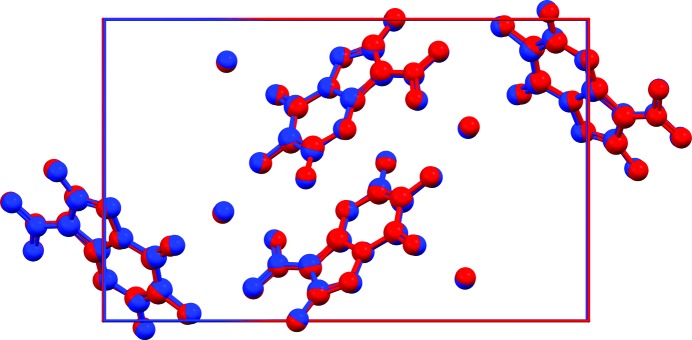
Overlay of the non-H atoms of LIPWEM (Chernyshev, Fitch *et al.*, 1999 ▸), experimental structure in red, energy-minimized structure (unit cell free) in blue, H atoms omitted for clarity. Although the protonation state is not correctly reproduced by the DFT-D minimizations, the positions of the non-H atoms are not affected. RMSCD = 0.062 Å.

The positions of the non-H atoms, however, are not affected at all: for LIPWEM, the r.m.s. value is only 0.062 Å (Fig. 6 ▸).

### 3.8. Space-group revisions   

No additional symmetry elements were found for any of the experimental structures. For the energy-minimized structures, some showed additional symmetry elements.

BUTYNE01 (Ibberson & Prager, 1995 ▸). The space group changes from *C*2/*m* to 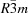 due to a minor shift of layers of molecules during energy minimization. The experimental structure was measured at *T* = 5 K, hence a temperature effect seems unlikely. There appears to be a subtle feature of the 2-butyne crystal structure that DFT-D is not able to reproduce. The published space group is correct.

**Figure 7:**
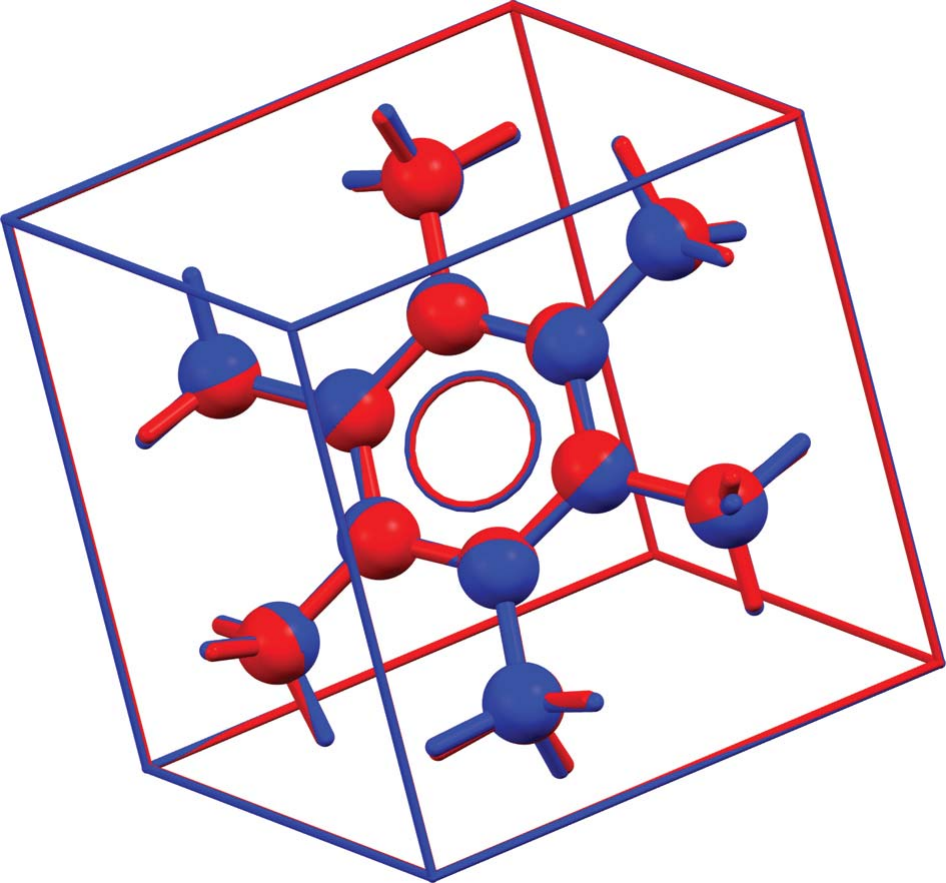
Overlay for HMBENZ06 (Stride, 2005 ▸) of the experimental structure in 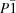 (red) and the energy-minimized structure (unit cell free) converted to 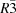 (rhombohedral setting) (blue). We conclude that the correct space group is 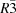. RMSCD = 0.029 Å.

HMBENZ06/HMBENZ07/HMBENZ08/HMBENZ09/MBENZ10/HMBENZ11 (Stride, 2005 ▸). The space group was changed from 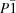, *Z*′ = ½ to 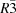, *Z*′ = 1/6. The near rhombohedral unit cell is noted explicitly in the original paper and refinement in the space group *R*3*m* was attempted but was unsuccessful. The experimental data are not available in the supporting information. Fig. 7 ▸ shows an overlay of the experimental structure and the energy-minimized structure with 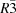 symmetry imposed. A crystal structure with the same unit cell and the space group 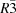 has been published before in an abstract [HMBENZ03 (Santarsiero *et al.*, 1985 ▸)], but without atomic coordinates. The correct space group is 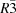. (We note that HMBENZ03 and HMBENZ06, measured at 19 and 5 K respectively, have a rhombohedral angle of ∼ 90.1°, whereas the energy-minimized structure has an angle of 89.99° and is therefore metrically cubic, but with the symmetry elements corresponding to a rhombohedral unit cell.)

QAMXUY01 (Mora *et al.*, 2005 ▸). The space group was changed from *Pn*2_1_ *a*, *Z*′ = 1 to *Pnma*, *Z*′ = ½. The systematic absences for *Pn*2_1_ *a* and *Pnma* are the same; nothing in the original paper indicates that the authors checked for higher symmetry. The experimental data are not available in the supporting information and the authors did not respond to a request for the data. The correct space group appears to be *Pnma*.

SILVAL/SILVAL02/SILVAL04/SILVAL05 (Helmholdt *et al.*, 2007 ▸). The space group was changed from *Pca*2_1_ to *Pcam* (a non-standard setting of *Pbcm*). The authors of the original paper refer to Raman data proving that the space group cannot be centrosymmetric, so the published space group must be assumed to be correct.

TMAPCL05/TMAPCL06 (Palacios *et al.*, 2003 ▸). The space group was changed from *P*2_1_2_1_2, *Z*′ = 1 to *Pbma*, *Z*′ = ½ (a non-standard setting of *Pbcm*). The authors of the original paper hesitate between 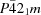, *Pbma* and *P*2_1_2_1_2; *P*2_1_2_1_2 is a maximal subgroup of 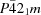 and *Pbma*. *P*2_1_2_1_2 was selected because it gave the structure with the lowest *R* value, but this is probably due to the fact that a lower space-group symmetry offers more degrees of freedom. For the powder data at *T* = 30 K (TMAPCL05) it is mentioned explicitly that *P*2_1_2_1_2 and *Pbma* led to the same model with the same *R* value, in which case the highest possible space-group symmetry should have been chosen. The correct space group appears to be *Pbma*.

ZZZUXA02/ZZZUXA03 (Damay *et al.*, 2008 ▸). The space group was changed from *P*2_1_, *Z*′ = 4 to *Pb*2_1_ *a*, *Z*′ = 2 (a non-standard setting of *Pca*2_1_). The compound undergoes a phase transition, and the authors of the original paper searched for a subgroup of *Pbnm* (a non-standard setting of *Pnma*) that allows for a doubling of the **a**-axis. The subgroup *Pb*2_1_ *m* (a non-standard setting of *Pmc*2_1_) was tried, but no satisfactory refinement could be obtained. *Pb*2_1_ *a* is a subgroup of *Pb*2_1_ *m*. The correct space group appears to be *Pb*2_1_ *a*.

ZZZVCO03 (Damay *et al.*, 2006 ▸)/ZZZVCO05 (Palatinus & Damay, 2009 ▸). ZZZVCO05 is a reinterpretation of ZZZVCO03, published in *P*4_1_, *Z*′ = 8, in the higher space group *P*4_1_2_1_2, *Z*′ = 4. The lattice energies differ by 0.008 kJ mol^−1^, which is within the numerical noise of the method. With deuterium atoms omitted, the energy-minimized ZZZVCO03 structure is indeed *P*4_1_2_1_2, but the experimental structure is *P*4_1_, even with deuterium atoms removed (within the tolerances used in this paper). Assuming that the methyl groups can rotate more or less freely, it appears that the correct space group is *P*4_1_2_1_2.

QIKZAN02 (Bekö *et al.*, 2014 ▸). The space group was changed from *P*2_1_2_1_2_1_, *Z*′ = 2 to *Pbca*, *Z*′ = 1. As discussed above, the positions of the H atoms are wrong, but the space group is correct as is discussed in some detail in the original paper.

None of the SX structures from the 2010 validation study had changed their space-group symmetry as a result of the energy minimization.

Space-group errors appear to be more common in structures from XRPD data than in those from SX data. Peak overlap hampers the space-group determination from the systematic absences, whereas the lower accuracy of the atomic coordinates makes it difficult to detect the space-group symmetry in real space. The higher space-group symmetry only becomes apparent after energy minimization, so the missed symmetry cannot be detected from the experimental structure directly; the energy minimization, however, always refers to 0 K and obscures temperature-dependent space-group changes.

## 4. Conclusion   

For 225 single-crystal structures, approximately three, or 1.3%, were incorrect; all three errors pertained to H-atom positions. For 215 XRPD structures, at least seven (FOMNAX, IJEKAJ, LAKKEO, LAQSON, SAXFED, XUDTIZ, YIRVOL) show an error in a non-H atom position, five (FANDOO, GOLTUW, MEXZOG, UKIRAI, QIKZAN02) have incorrectly placed H atoms and five (HMBENZ, QAMXUY, TMAPCL, ZZZUXA, ZZZVCO) were almost certainly determined in a subgroup of the actual space group. Two structures are almost certainly wrong (QAMXUY, VANGAT) but no satisfactory model could be found. In total, 19/215 = 8.8% of the crystal structures determined from XRPD are demonstrably in error. Reassuringly, at least for those XRPD structures that are published in IUCr journals, the errors that are present in the published crystal structures are virtually all minor: minor space-group revisions, exchanges of *e.g.* C and N, and ambiguities involving H atoms. In some cases the possible ambiguities had already been spotted by the original authors, but no instrument existed to resolve the ambiguity; we have shown here that Dispersion-corrected Density Functional Theory (DFT-D) calculations provide an independent source of structural information about organic crystal structures and as such are ideally suited to boost the reduced information content of an experimental powder diffraction pattern.

Based on the DFT-D calculations, three crystal structures (BICCIZ01, WUBDOM, zb5033_FormX) could be re-refined using the original powder diffraction data to give substantially more precise atomic coordinates.

Preferred orientation is the greatest source of problems and uncertainties. Clearly, the presence of preferred orientation should be regarded with much more suspicion than is currently the norm.

## Supplementary Material

Revised experimental structures. DOI: 10.1107/S2052520614022902/kd5080sup1.zip

Supplementary Material for revised structures. DOI: 10.1107/S2052520614022902/kd5080sup2.pdf

Energy-minimised structures. DOI: 10.1107/S2052520614022902/kd5080sup3.zip

## References

[bb1] Allen, F. H. (2002). *Acta Cryst.* B**58**, 380–388. 10.1107/s0108768102003890 12037359

[bb179] Arnott, S. & Dover, S. D. (1968). *Acta Cryst.* B**24**, 599–601. 10.1107/s056774086800289x 5756983

[bb2] Avila, E. E., Mora, A. J., Delgado, G. E., Contreras, R. R., Fitch, A. N. & Brunelli, M. (2008). *Acta Cryst.* B**64**, 217–222. 10.1107/S0108768108000190 18369293

[bb147] Bekö, S. L., Alig, E., Schmidt, M. U. & Van de Streek, J. (2014). *IUCrJ*, **1** 61–73. 10.1107/S2052252513026511 PMC4104965 25075320

[bb3] Bekö, S. L., Thoms, S. D., Brüning, J., Alig, E., Van de Streek, J., Lakatos, A., Glaubitz, C. & Schmidt, M. U. (2010). *Z. Kristallogr.* **225**, 382–387.

[bb118] Bhuvanesh, N. S. P., Reibenspies, J. H., Zhang, Y. & Lee, P. L. (2005). *J. Appl. Cryst.* **38**, 632–638.

[bb4] Blanton, T. N., Rajeswaran, M., Stephens, P. W., Whitcomb, D. R., Misture, S. T. & Kaduk, J. A. (2011). *Powder Diffr.* **26**, 313–320.

[bb34] Bortolotti, M., Lonardelli, I. & Pepponi, G. (2011). *Acta Cryst.* B**67**, 357–364. 10.1107/S0108768111021653 21775814

[bb173] Brenner, S., McCusker, L. B. & Baerlocher, C. (2002). *J. Appl. Cryst.* **35**, 243–252.

[bb84] Brunelli, M. & Fitch, A. N. (2003). *J. Synchrotron Rad.* **10**, 337–339. 10.1107/s0909049503007969 12824935

[bb5] Bruno, I. J., Cole, J. C., Kessler, M., Luo, J., Motherwell, W. D. S., Purkis, L. H., Smith, B. R., Taylor, R., Cooper, R. I., Harris, S. E. & Orpen, A. G. (2004). *J. Chem. Inf. Comput. Sci.* **44**, 2133–2144. 10.1021/ci049780b 15554684

[bb252] Buchsbaum, C. & Schmidt, M. U. (2007). *Acta Cryst.* B**63**, 926–932. 10.1107/S0108768107050823 18004047

[bb160] Burley, J. C. (2005). *Acta Cryst.* B**61**, 710–716. 10.1107/S0108768105025991 16306678

[bb66] Bushmarinov, I. S., Dmitrienko, A. O., Korlyukov, A. A. & Antipin, M. Yu. (2012). *J. Appl. Cryst.* **45**, 1187–1197.

[bb115] Chernyshev, V. V., Fitch, A. N., Sonneveld, E. J., Kurbakov, A. I., Makarov, V. A. & Tafeenko, V. A. (1999). *Acta Cryst.* B**55**, 554–562. 10.1107/s010876819900213x 10927397

[bb186] Chernyshev, V. V., Machula, A. A., Kukushkin, S. Y. & Velikodny, Y. A. (2009). *Acta Cryst.* E**65**, o2020–o2021. 10.1107/S1600536809029353 PMC2977435 21583690

[bb181] Chernyshev, V. V., Paseshnichenko, K. A., Makarov, V. A., Sonneveld, E. J. & Schenk, H. (2001). *Acta Cryst.* C**57**, 72–75. 10.1107/s0108270100013457 11173404

[bb127] Chernyshev, V. V., Shkavrov, S. V., Paseshnichenko, K. A., Puryaeva, T. P. & Velikodny, Y. A. (2013). *Acta Cryst.* C**69**, 263–266. 10.1107/S0108270113003089 23459352

[bb191] Chernyshev, V. V., Tafeenko, V. A., Ryabova, S. Y., Sonneveld, E. J. & Schenk, H. (2001). *Acta Cryst.* C**57**, 982–984. 10.1107/s0108270101008356 11498634

[bb97] Chernyshev, V. V., Yatsenko, A. V., Kuvshinov, A. M. & Shevelev, S. A. (2002). *J. Appl. Cryst.* **35**, 669–673.

[bb76] Chernyshev, V. V., Yatsenko, A. V., Tafeenko, V. A., Makarov, V. A., Sonneveld, E. J. & Schenk, H. (1999). *Acta Cryst.* C**55**, 1099–1101.

[bb79] Clark, J. C., McLaughlin, M. L. & Fronczek, F. R. (2003). *Acta Cryst.* E**59**, o2005–o2006.

[bb6] Clark, S. J., Segall, M. D., Pickard, C. J., Hasnip, P. J., Probert, M. J., Refson, K. & Payne, M. C. (2005). *Z. Kristallogr.* **220**, 567–570.

[bb7] Coelho, A. A. (2012). *TOPAS-Academic*, Version 5. Coelho Software, Brisbane.

[bb73] David, W. I. F., Ibberson, R. M., Cox, S. F. J. & Wood, P. T. (2006). *Acta Cryst.* B**62**, 953–959. 10.1107/S0108768106039309 17108646

[bb8] David, W. I. F., Shankland, K., Van de Streek, J., Pidcock, E., Motherwell, W. D. S. & Cole, J. C. (2006). *J. Appl. Cryst.* **39**, 910–915.

[bb194] Day, G. M. *et al.* (2009). *Acta Cryst.* B**65**, 107–125. 10.1107/S0108768109004066 19299868

[bb199] Damay, F., Carretero-Genevrier, A., Cousson, A., Van Beek, W., Rodriguez-Carvajal, J. & Fillaux, F. (2006). *Acta Cryst.* B**62**, 627–633. 10.1107/S0108768106011608 16840812

[bb198] Damay, F., Rodríguez-Carvajal, J., André, D., Dunstetter, F. & Szwarc, H. (2008). *Acta Cryst.* B**64**, 589–595. 10.1107/S0108768108015103 18799847

[bb9] Deringer, V. L., Hoepfner, V. & Dronskowski, R. (2012). *Cryst. Growth Des.* **12**, 1014–1021.

[bb132] Derollez, P., Correia, N. T., Danède, F., Capet, F., Affouard, F., Lefebvre, J. & Descamps, M. (2005). *Acta Cryst.* B**61**, 329–334. 10.1107/S010876810500546X 15914898

[bb95] Derollez, P., Dudognon, E., Affouard, F., Danède, F., Correia, N. T. & Descamps, M. (2010). *Acta Cryst.* B**66**, 76–80. 10.1107/S0108768109047363 20101086

[bb125] Derollez, P., Hédoux, A., Guinet, Y., Danède, F. & Paccou, L. (2013). *Acta Cryst.* B**69**, 195–202. 10.1107/S2052519213004843 23719706

[bb77] Dinnebier, R. E., Dollase, W. A., Helluy, X., Kümmerlen, J., Sebald, A., Schmidt, M. U., Pagola, S., Stephens, P. W. & van Smaalen, S. (1999). *Acta Cryst.* B**55**, 1014–1029. 10.1107/s0108768199006126 10927444

[bb10] Dollase, W. A. (1986). *J. Appl. Cryst.* **19**, 267–272.

[bb11] Florence, A. J., Bardin, J., Johnston, B., Shankland, N., Griffin, T. A. N. & Shankland, K. (2009). *Z. Kristallogr.* **30**, 215–220.

[bb70] Florence, A. J., Baumgartner, B., Weston, C., Shankland, N., Kennedy, A. R., Shankland, K. & David, W. I. F. (2003). *J. Pharm. Sci.* **92**, 1930–1938. 10.1002/jps.10459 12950010

[bb12] Grimme, S. (2006). *J. Comput. Chem.* **27**, 1787–1799. 10.1002/jcc.20495 16955487

[bb13] Grimme, S., Antony, J., Ehrlich, S. & Krieg, H. (2010). *J. Chem. Phys.* **132**, 154104-1–154104-19. 10.1063/1.3382344 20423165

[bb56] Grzesiak, A. L. & Matzger, A. J. (2007). *J. Pharm. Sci.* **96**, 2978–2986. 10.1002/jps.20954 PMC2581769 17567888

[bb109] Guiry, K. P., Coles, S. J., Moynihan, H. A. & Lawrence, S. E. (2008). *Cryst. Growth Des.* **8**, 3927–3934.

[bb36] Gunn, E., Guzei, I. A., Cai, T. & Yu, L. (2012). *Cryst. Growth Des.* **12**, 2037–2043.

[bb67] Halasz, I., Dinnebier, R., Chiodo, T. & Saxell, H. (2012). *Acta Cryst.* B**68**, 661–666. 10.1107/S0108768112036191 23165602

[bb172] Hangan, A., Borodi, G., Filip, X., Tripon, C., Morari, C., Oprean, L. & Filip, C. (2010). *Acta Cryst.* B**66**, 615–621. 10.1107/S0108768110039327 21099024

[bb128] Helmholdt, R. B., Peschar, R. & Schenk, H. (2002). *Acta Cryst.* B**58**, 134–139. 10.1107/s0108768101016330 11818661

[bb164] Helmholdt, R. B., Sonneveld, E. J., Vande Velde, C. M. L., Blockhuys, F., Lenstra, A. T. H., Geise, H. J. & Peschar, R. (2007). *Acta Cryst.* B**63**, 783–790. 10.1107/S010876810703652X 17873447

[bb142] Hernandez, O., Hédoux, A., Lefebvre, J., Guinet, Y., Descamps, M., Papoular, R. & Masson, O. (2002). *J. Appl. Cryst.* **35**, 212–219.

[bb41] Ibberson, R. M. & Prager, M. (1995). *Acta Cryst.* B**51**, 71–76.

[bb182] Ivashevskaja, S. N., Aleshina, L. A., Andreev, V. P., Nizhnik, Y. P. & Chernyshev, V. V. (2002). *Acta Cryst.* E**58**, o920–o922. 10.1107/s0108270102005292 11983973

[bb98] Ivashevskaja, S. N., Aleshina, L. A., Andreev, V. P., Nizhnik, Y. P., Chernyshev, V. V. & Schenk, H. (2003). *Acta Cryst.* E**59**, o1006–o1008. 10.1107/s0108270102005292 11983973

[bb195] Jaroń, T. & Grochala, W. (2011). *Acta Cryst.* E**67**, o2171. 10.1107/S1600536811029291 PMC3213606 22091183

[bb137] Johnston, A., Florence, A. J., Shankland, K., Markvardsen, A., Shankland, N., Steele, G. & Cosgrove, S. D. (2004). *Acta Cryst.* E**60**, o1751–o1753.

[bb120] Kaduk, J. A. (2000). *Acta Cryst.* B**56**, 474–485. 10.1107/s0108768199014718 10877356

[bb166] Kaduk, J. A. & Golab, J. T. (1999). *Acta Cryst.* B**55**, 85–94. 10.1107/s0108768198008945 10927342

[bb60] Krebs, F. C., Jørgensen, M., Lebech, B. & Frydenvang, K. (2001). *J. Appl. Cryst.* **34**, 203–207.

[bb14] Kresse, G. & Furthmüller, J. (1996*a*). *Comput. Mater. Sci*, **6**, 15–50.

[bb15] Kresse, G. & Furthmüller, J. (1996*b*). *Phys. Rev. B*, **54**, 11169–11186. 10.1103/physrevb.54.11169 9984901

[bb40] Kutzke, H., Klapper, H., Hammond, R. B. & Roberts, K. J. (2000). *Acta Cryst.* B**56**, 486–496. 10.1107/s0108768100000355 10877357

[bb92] Lasocha, W., Gaweł, B., Rafalska-Lasocha, A., Pawłowski, M., Talik, P. & Paszkowicz, W. (2010). *J. Appl. Cryst.* **43**, 163–167.

[bb108] Lefebvre, J., Willart, J.-F., Caron, V., Lefort, R., Affouard, F. & Danède, F. (2005). *Acta Cryst.* B**61**, 455–463. 10.1107/S0108768105017064 16041096

[bb136] Lehmann, A., Luger, P., Lehmann, C. W. & Ibberson, R. M. (1994). *Acta Cryst.* B**50**, 344–348.

[bb16] Li, X., Bond, A. D., Johansson, K. E. & Van de Streek, J. (2014). *Acta Cryst.* C**70**, 784–789. 10.1107/S2053229614015356 PMC4174016 25093360

[bb55] Llinàs, A., Box, K. J., Burley, J. C., Glen, R. C. & Goodman, J. M. (2007). *J. Appl. Cryst.* **40**, 379–381.

[bb161] Llinàs, A., Fábián, L., Burley, J. C., Van de Streek, J. & Goodman, J. M. (2006). *Acta Cryst.* E**62**, o4196–o4199.

[bb192] Logacheva, N. M., Puryaeva, T. P., Tsivadze, A. Y., Velikodny, Y. A. & Chernyshev, V. V. (2008). *Acta Cryst.* E**64**, o225. 10.1107/S1600536807064999 PMC2915286 21200792

[bb17] Macrae, C. F., Bruno, I. J., Chisholm, J. A., Edgington, P. R., McCabe, P., Pidcock, E., Rodriguez-Monge, L., Taylor, R., Van de Streek, J. & Wood, P. A. (2008). *J. Appl. Cryst.* **41**, 466–470.

[bb162] Mangwala Kimpende, P. & Van Meervelt, L. (2010). *Acta Cryst.* E**66**, o2353–o2354. 10.1107/S1600536810031806 PMC3007921 21588696

[bb156] Mechelen, J. B. van, Goubitz, K., Pop, M., Peschar, R. & Schenk, H. (2008). *Acta Cryst.* B**64**, 771–779. 10.1107/S0108768108031601 19029706

[bb101] Mechelen, J. B. van, Peschar, R. & Schenk, H. (2006*a*). *Acta Cryst.* B**62**, 1121–1130. 10.1107/S0108768106037074 17108667

[bb102] Mechelen, J. B. van, Peschar, R. & Schenk, H. (2006*b*). *Acta Cryst.* B**62**, 1131–1138. 10.1107/S0108768106037086 17108668

[bb48] Meriles, C. A., Schneider, J. F., Mascarenhas, Y. P. & Brunetti, A. H. (2000). *J. Appl. Cryst.* **33**, 71–81.

[bb117] Moggach, S. A., Marshall, W. G. & Parsons, S. (2006). *Acta Cryst.* B**62**, 815–825. 10.1107/S010876810601799X 16983163

[bb144] Mora, A. J., Avila, E. E., Delgado, G. E., Fitch, A. N. & Brunelli, M. (2005). *Acta Cryst.* B**61**, 96–102. 10.1107/S0108768104031738 15659862

[bb163] Muangsin, N., Prajuabsook, M., Chimsook, P., Chantarasiri, N., Siraleartmukul, K., Chaichit, N. & Hannongbua, S. (2004). *J. Appl. Cryst.* **37**, 288–294.

[bb18] Naelapää, K., Van de Streek, J., Rantanen, J. & Bond, A. D. (2012). *J. Pharm. Sci.* **101**, 4214–4219. 10.1002/jps.23287 22886472

[bb19] Neumann, M. A. (2013). *GRACE*, Version 2.1, http://www.avmatsim.eu. Avant-garde Materials Simulation SARL, France.

[bb21] Neumann, M. A. & Perrin, M.-A. (2005). *J. Phys. Chem. B*, **109**, 15531–15541. 10.1021/jp050121r 16852970

[bb22] Neumann, M. A., Tedesco, C., Destri, S., Ferro, D. R. & Porzio, W. (2002). *J. Appl. Cryst.* **35**, 296–303.

[bb111] Noguchi, S., Fujiki, S., Iwao, Y., Miura, K. & Itai, S. (2012). *Acta Cryst.* E**68**, o667–o668. 10.1107/S1600536812005090 PMC3295456 22412567

[bb131] Noguchi, S., Miura, K., Fujiki, S., Iwao, Y. & Itai, S. (2012). *Acta Cryst.* C**68**, o41–o44. 10.1107/S0108270111054230 22307251

[bb47] Ochando, L. E., Rius, J., Louër, D., Claramunt, R. M., Lopez, C., Elguero, J. & Amigó, J. M. (1997). *Acta Cryst.* B**53**, 939–944.

[bb169] Palacios, E., Burriel, R. & Ferloni, P. (2003). *Acta Cryst.* B**59**, 625–633. 10.1107/s0108768103019013 14586083

[bb200] Palatinus, L. & Damay, F. (2009). *Acta Cryst.* B**65**, 784–786. 10.1107/S0108768109042608 19923707

[bb71] Papoular, R. J., Allouchi, H., Chagnes, A., Dzyabchenko, A., Carré, B., Lemordant, D. & Agafonov, V. (2005). *Acta Cryst.* B**61**, 312–320. 10.1107/S0108768105005392 15914896

[bb74] Pawley, G. S. & Whitley, E. (1988). *Acta Cryst.* C**44**, 1249–1251.

[bb23] Perdew, J. P., Burke, K. & Ernzerhof, M. (1996). *Phys. Rev. Lett.* **77**, 3865–3868. 10.1103/PhysRevLett.77.3865 10062328

[bb251] Pop, M. M., Goubitz, K., Borodi, G., Bogdan, M., De Ridder, D. J. A., Peschar, R. & Schenk, H. (2002). *Acta Cryst.* B**58**, 1036–1043. 10.1107/s010876810201947x 12456984

[bb135] Putkonen, M.-L., Feld, R., Vettier, C. & Lehmann, M. S. (1985). *Acta Cryst.* B**41**, 77–79.

[bb250] Rácz, C.-P., Borodi, G., Pop, M. M., Kacso, I., Sánta, S. & Tomoaia-Cotisel, M. (2012). *Acta Cryst.* B**68**, 164–170. 10.1107/S0108768112004284 22436915

[bb46] Reck, G., Kretschmer, R.-G., Kutschabsky, L. & Pritzkow, W. (1988). *Acta Cryst.* A**44**, 417–421.

[bb39] Rohlicek, J., Husak, M., Gavenda, A., Jegorov, A., Kratochvil, B. & Fitch, A. (2009). *Acta Cryst.* E**65**, o1325–o1326. 10.1107/S1600536809017905 PMC2969674 21583180

[bb65] Rukiah, M., Lefebvre, J., Descamps, M., Hemon, S. & Dzyabchenko, A. (2004). *J. Appl. Cryst.* **37**, 464–471.

[bb75] Rukiah, M., Lefebvre, J., Hernandez, O., van Beek, W. & Serpelloni, M. (2004). *J. Appl. Cryst.* **37**, 766–772.

[bb114] Rybakov, V. B., Babaev, E. V., Sonneveld, E. J., Zhukov, S. G. & Chernyshev, V. V. (2007). *Acta Cryst.* E**63**, o1861–o1863.

[bb253] Santarsiero, B. D., Bronikowski, M. J. & Samson, S. O. (1985). *ACA Abstr. Papers (Winter)*, **13**, 55.

[bb158] Seijas, L. E., Mora, A. J., Delgado, G. E., López-Carrasquero, F., Báez, M. E., Brunelli, M. & Fitch, A. N. (2009). *Acta Cryst.* B**65**, 724–730. 10.1107/S0108768109036386 19923701

[bb32] Sergeev, G. B., Sergeev, B. M., Morosov, Y. N. & Chernyshev, V. V. (2010). *Acta Cryst.* E**66**, o2623. 10.1107/S1600536810037402 PMC2983154 21587597

[bb69] Shankland, K., McBride, L., David, W. I. F., Shankland, N. & Steele, G. (2002). *J. Appl. Cryst.* **35**, 443–454.

[bb24] Sholl, D. S. & Steckel, J. A. (2009). *Density Functional Theory – A Practical Introduction.* New Jersey: John Wiley and Sons Inc.

[bb25] Smrčok, L. (2012). *Uniting Electron Crystallography and Powder Diffraction*, edited by U. Kolb, K. Shankland, L. Meshi, A. Avilov & W. I. F. David. Dordrecht: Springer Science + Business Media.

[bb49] Stone, K. H., Lapidus, S. H. & Stephens, P. W. (2009). *J. Appl. Cryst.* **42**, 385–391.

[bb86] Stride, J. A. (2005). *Acta Cryst.* B**61**, 200–206. 10.1107/S0108768104034007 15772453

[bb103] Tanahashi, Y., Nakamura, H., Yamazaki, S., Kojima, Y., Saito, H., Ida, T. & Toraya, H. (2001). *Acta Cryst.* B**57**, 184–189. 10.1107/s0108768100018905 11262433

[bb78] Tremayne, M., MacLean, E. J., Tang, C. C. & Glidewell, C. (1999). *Acta Cryst.* B**55**, 1068–1074. 10.1107/s0108768199006060 10927448

[bb187] Tremayne, M., Seaton, C. C. & Glidewell, C. (2002). *Acta Cryst.* B**58**, 823–834. 10.1107/s0108768102011928 12324695

[bb27] Van de Streek, J. (2006). *Acta Cryst.* B**62**, 567–579. 10.1107/S0108768106019677 16840806

[bb28] Van de Streek, J. (2012). *Acta Cryst.* C**68**, o369–o372. 10.1107/S0108270112035536 22935507

[bb26] Van de Streek, J. & Neumann, M. A. (2010). *Acta Cryst.* B**66**, 544–558. 10.1107/S0108768110031873 PMC2940256 20841921

[bb184] Van Langevelde, A., Van Malssen, K., Driessen, R., Goubitz, K., Hollander, F., Peschar, R., Zwart, P. & Schenk, H. (2000). *Acta Cryst.* B**56**, 1103–1111. 10.1107/s0108768100009927 11099979

[bb80] Vella-Zarb, L. & Dinnebier, R. E. (2012). *Acta Cryst.* B**68**, 204–208. 10.1107/S0108768112008592 22436919

[bb50] Wilding, N. B., Crain, J., Hatton, P. D. & Bushnell-Wye, G. (1993). *Acta Cryst.* B**49**, 320–328.

[bb89] Wunschel, M., Dinnebier, R. E., Carlson, S., Bernatowicz, P. & van Smaalen, S. (2003). *Acta Cryst.* B**59**, 60–71. 10.1107/s0108768102021791 12554973

[bb188] Zhou, T., Zhang, Q., Chen, G. & Zhou, Z. (2004). *Acta Cryst.* E**60**, o1767–o1768.

[bb29] Zvirgzdins, A., Mishnev, A. & Actins, A. (2014). *Acta Cryst.* B**70**, 342–346. 10.1107/S2052520614001140 24675603

