# Supplementary material for: Validation of molecular crystal structures from powder diffraction data with dispersion-corrected density functional theory (DFT-D)
Source: Acta Crystallogr B Struct Sci Cryst Eng Mater. 2014 Dec 1;70(Pt 6):1020–32. doi: 10.1107/S2052520614022902 (PMC4468513; doi:10.1107/S2052520614022902)
Supplement: Supplementary file 2 [file b-70-01020-sup2.pdf]

Rietveld refinements

Experimental data in red, calculated pattern in blue, difference curve in black, tickmarks in blue.

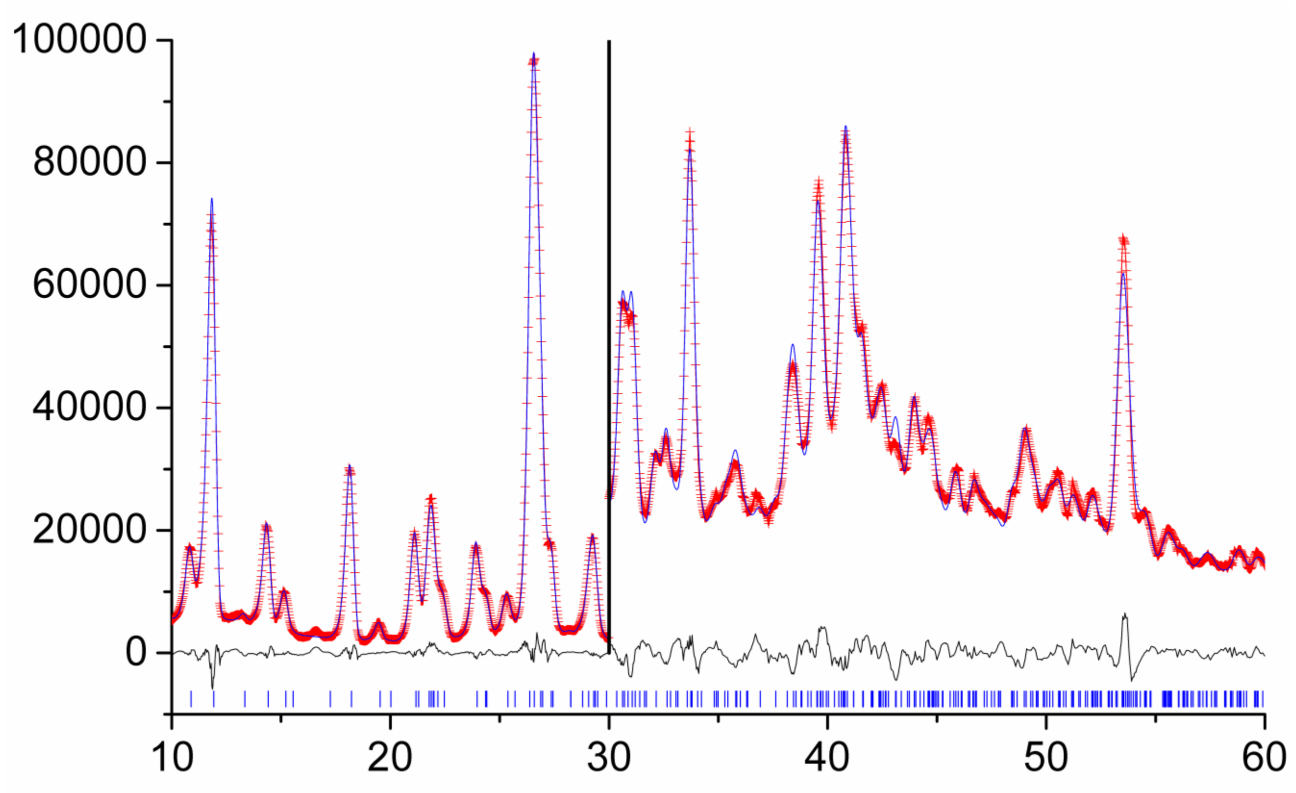

Figure S1. WUBDOM.

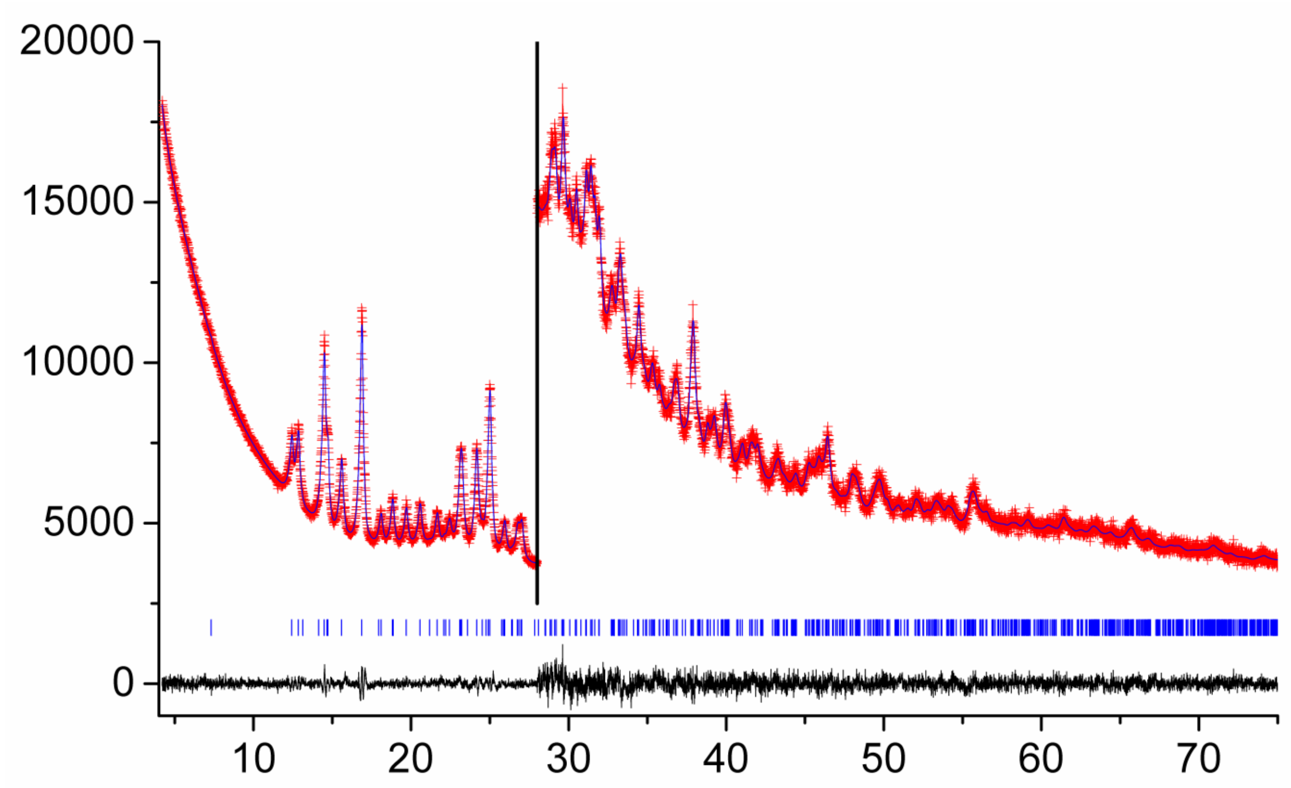

Figure S2. zb5033 Form X.

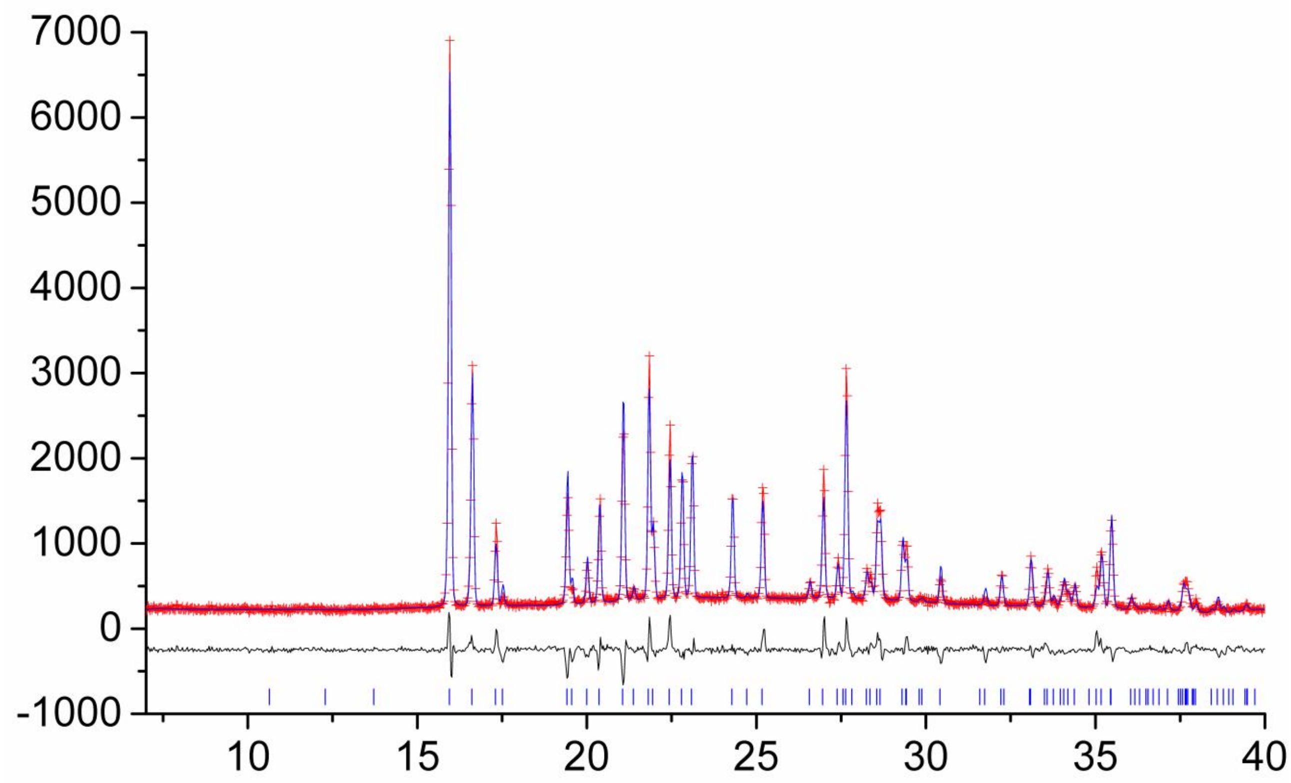

Figure S3. FOMNAX.

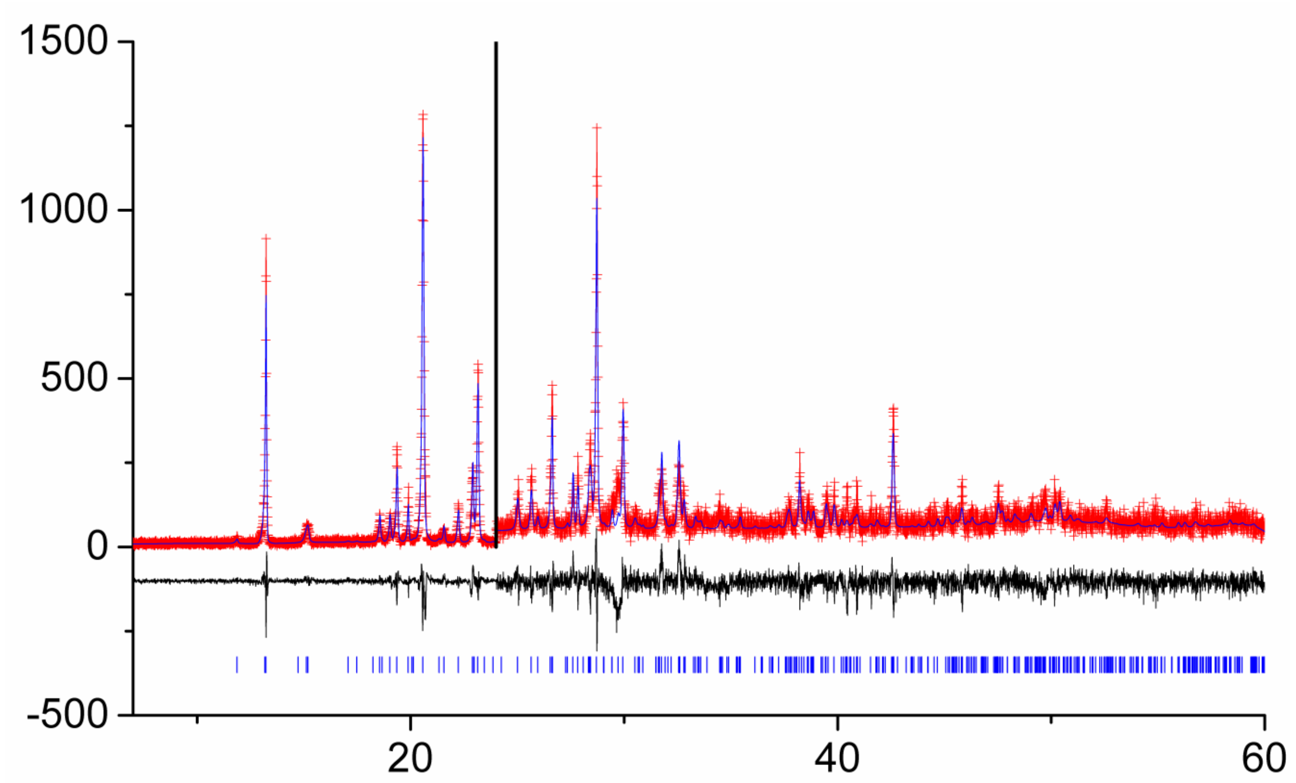

Figure S4. IJEKAJ.

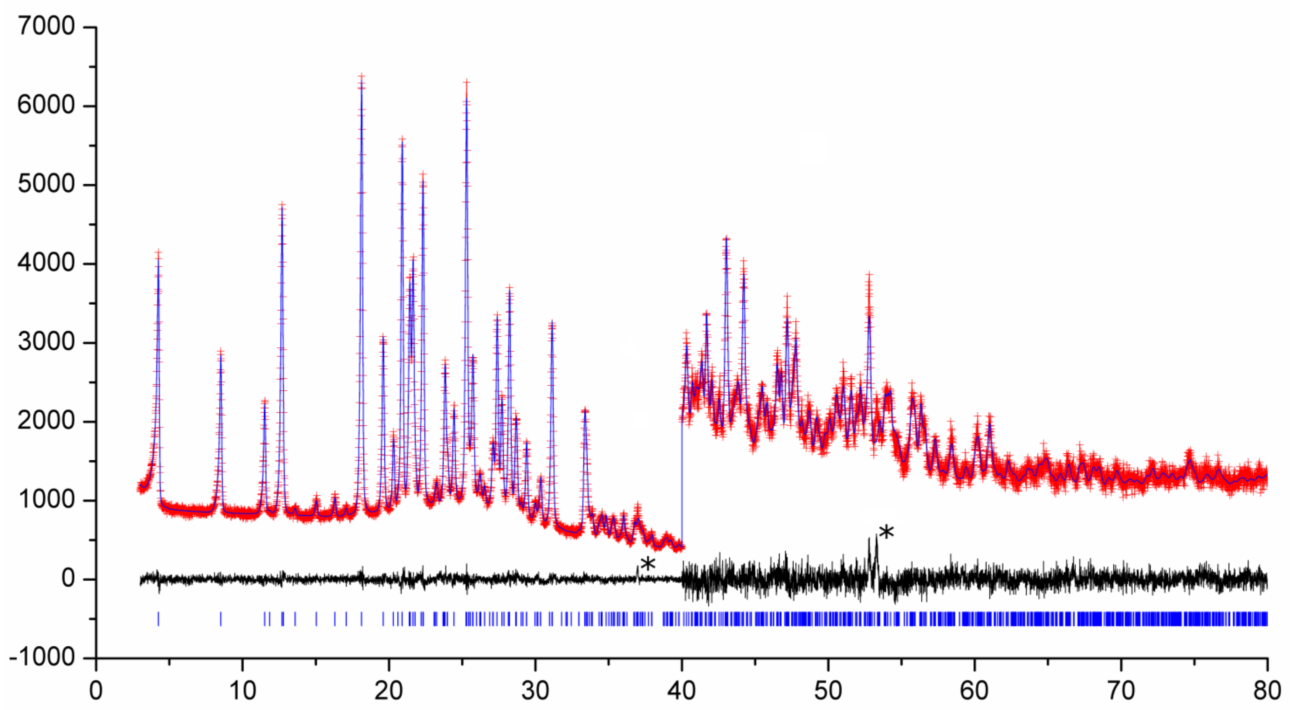

Figure S5. SAXFED. The two residual peaks indicated by asterisks can be assigned to NaCl.

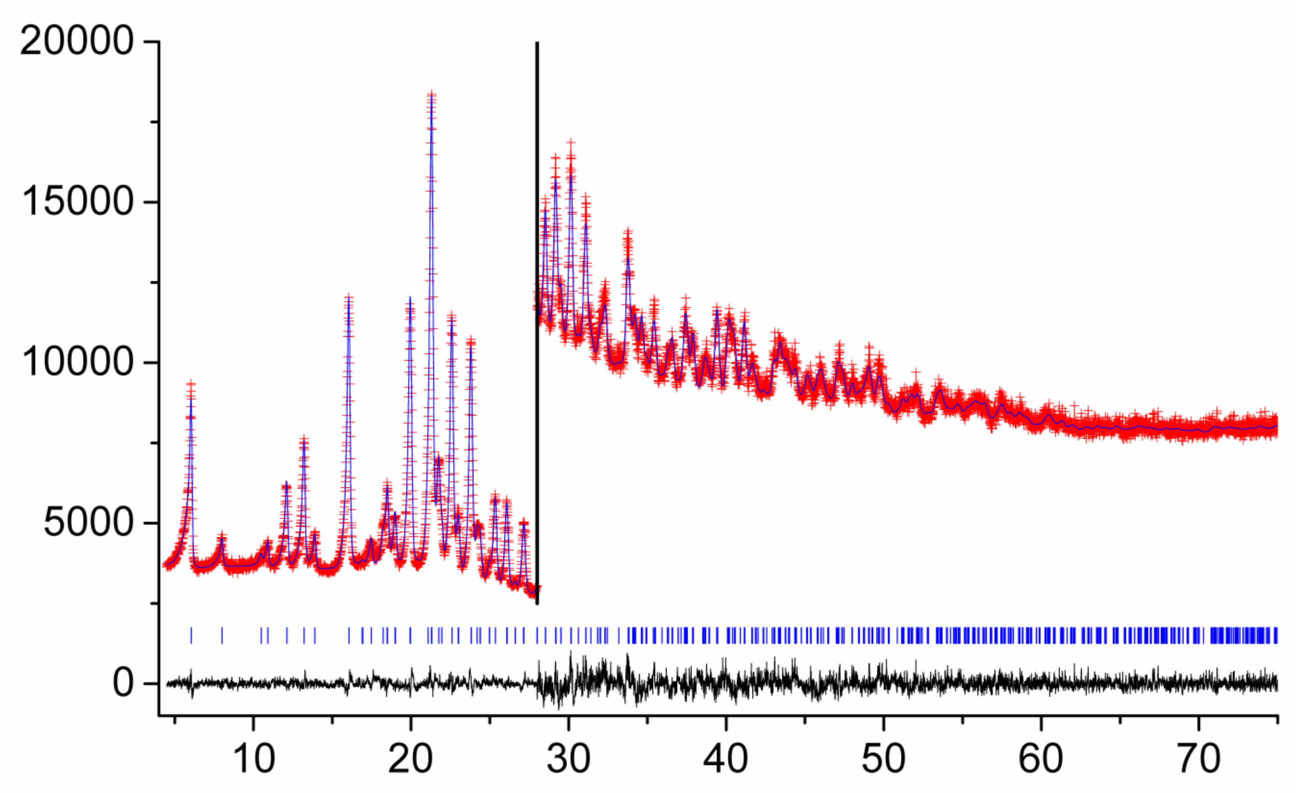

Figure S6. YIRVOL.

Rietveld refinement results

	WUBDOM	zb5033 X	FOMNAX	IJEKAJ	SAXFED	YIRVOL
Formula	$C_9H_7NO{\cdot}2H_2O$	$C_{12}H_{17}N_2S^+Cl^-$	$C_4H_6O_2$	$C_{15}H_{16}N_2O$	$C_{21}H_{27}N_5O_4S$	$C_{29}H_{29}N_3O_6$
a	9.4704(12)	12.1132(19)	10.1372(4)	26.838(2)	9.1401(2)	58.324(4)
b	16.240(2)	8.3808(11)	10.2395(4)	7.7598(5)	24.2640(6)	33.6850(17)
c	6.9009(7)	13.522(2)	8.3201(3)	6.0806(6)	5.17462(11)	5.3163(2)
α	90	90	90	90	93.126(2)	90
β	118.294(5)	94.938(4)	93.2889(17)	94.049(5)	101.1550(17)	90
γ	90	90	90	90	83.4798(12)	90
V	934.5(2)	1367.6(3)	862.20(5)	1263.15(18)	1118.02(5)	10444.7(9)
Space group	$P2_1/n$	$P2_1/c$	$P2_1/a$	$P2_1/n$	*P*-1	*Fdd*2
Z/Z'	4/1	4/1	8/2	4/1	2/1	16/1
R'_{wp}	7.83	12.8	19.4	38.1	9.40	9.36
R_{wp}	4.93	2.04	9.29	23.9	3.82	2.26
X^2	11.6	1.23	3.92	1.59	1.11	1.61
PO, direction	(010)	-	(011)	(100)	-	-
PO, *r*	0.93	-	1.77	0.69	-	-

Explanatory notes to the structures in the ESI

Structures listed under "Experiment" are reinterpretations of previously published crystal structures from XRPD and are intended for inclusion in *e.g*. the Cambridge Structural Database. All unit-cell parameters and atomic coordinates for non-H atoms are based on the experimental data, the experimental unit cell and the experimental atomic coordinates as published. The positions of all H atoms are the result of a DFT-D energy-minimisation with the unit cell and positions of the non-H atoms kept fixed. Where a single-crystal (SX) structure is known, that structure is considered to be the definite structure. Structures listed under "Energy-minimisation" are the corresponding energy-minimised structures with the unit cell free; these structures, with file names ending in *_mi_ucf.cif, are the result of an energy-minimisation and should not be incorporated in *e.g*. the Cambridge Structural Database.

Re-refined structures

The unit cell and the non-H atoms were obtained by Rietveld refinement against the published XRPD data of the original publications, the positions of all H atoms are the result of a DFT-D energy-minimisation with the unit cell and positions of the non-H atoms kept fixed.

Experiment	Energy-minimisation
[BICCIZ03, SX]	BICCIZ01_mi_ucf.cif[a]
[EPHEDH02, SX]	EPHEDH03_mi_ucf.cif[a]
WUBDOM_rerefined.cif	WUBDOM_mi_ucf.cif[a]
zb5033_FormX_rerefined.cif	zb5033_FormX_mi_ucf.cif[a]

[a] The energy-minimised structures of the low-quality and the high-quality structures are the same.

Corrected structures

The unit cell and the non-H atoms were obtained by Rietveld refinement against the published XRPD data of the original publications, the positions of all H atoms are the result of a DFT-D energy-minimisation with the unit cell and positions of the non-H atoms kept fixed.

Experiment	Energy-minimisation
[FOMNAX]	FOMNAX_corrected_mi_ucf.cif[a]
FOMNAX_corrected.cif	FOMNAX_corrected_mi_ucf.cif[a]
[IJEKAJ]	IJEKAJ_org_mi_ucf.cif
IJEKAJ_corrected.cif	IJEKAJ_corrected_mi_ucf.cif
[LAKKEO][b]	LAKKEO_org_mi_ucf.cif[b]
LAKKEO01_Hcorrected.cif[b]	LAKKEO01_Hcorrected_mi_ucf.cif[b]
[LAQSON]	-
[LAQSON01]	LAQSON01_mi_ucf.cif
[SAXFED]	SAXFED_org_mi_ucf.cif
SAXFED_corrected.cif	SAXFED_corrected_mi_ucf.cif
[XUDTIZ]	XUDTIZ_org_mi_ucf.cif
[XUDTIZ01, SX]	XUDTIZ_corrected_mi_ucf.cif
[YIRVOL]	YIRVOL_org_mi_ucf.cif
YIRVOL_corrected.cif	YIRVOL_corrected_mi_ucf.cif

[a] The energy-minimised structures of the original and the corrected structure are the same.

[b] LAKKEO is an XRPD structure with an incorrectly positioned C atom; LAKKEO01 is an SX structure with incorrectly positioned H atoms.

Added H atoms

The unit cell and the non-H atoms are taken from the original publications, the positions of all H atoms are the result of a DFT-D energy-minimisation with the unit cell and positions of the non-H atoms kept fixed.

Experiment	Energy-minimisation
[BPHENO03, SX]	BPHENO02_mi_ucf.cif
CEWVOP01_Hadded.cif	CEWVOP01_Hadded_mi_ucf.cif
CNITBZ01_Hadded.cif	CNITBZ01_Hadded_mi_ucf.cif
[CYCHEX, SX]	CYCHEX07_mi_ucf.cif
[GUFQED01, SX]	GUFQED_mi_ucf.cif
[a]	HUWSAU_mi_ucf.cif
QIBQIB01_Hadded.cif	QIBQIB01_Hadded_mi_ucf.cif
[SIKLIH07, SX]	SIKLIH06_mi_ucf.cif
[a]	WIMWOE_Hadded_mi_ucf.cif
WOVTEG_Hadded.cif	WOVTEG_Hadded_mi_ucf.cif
XARNAG_Hadded.cif	XARNAG_Hadded_mi_ucf.cif

[a] The structure is of low quality and would hardly be improved by the addition of H atoms.

Corrected H atoms

The unit cell and the non-H atoms are taken from the original publications, the positions of all H atoms are the result of a DFT-D energy-minimisation with the unit cell and positions of the non-H atoms kept fixed.

Experiment	Energy-minimisation
[QIKZAN02]	QIKZAN02_org_mi_ucf.cif
QIKZAN02_Hcorrected.cif	QIKZAN02_Hcorrected_mi_ucf.cif
[FANDOO]	FANDOO_Hcorrected_mi_ucf.cif[a]
FANDOO_Hcorrected	FANDOO_Hcorrected_mi_ucf.cif[a]
[FOGVIG02]	FOGVIG02_mi_ucf.cif[a]
[FOGVIG03, SX]	FOGVIG02_mi_ucf.cif[a]
[GOLTUW]	GOLTUW_org_mi_ucf.cif
GOLTUW_Hcorrected.cif	GOLTUW_Hcorrected_mi_ucf.cif
[LAKKEO][b]	LAKKEO_org_mi_ucf.cif[b]
LAKKEO01_Hcorrected.cif[b]	LAKKEO01_Hcorrected_mi_ucf.cif[b]
[LIPVUB]	LIPVUB_Hcorrected_mi_ucf.cif[a]
LIPVUB_Hcorrected.cif	LIPVUB_Hcorrected_mi_ucf.cif[a]
[MEXZOG]	MEXZOG_org_mi_ucf.cif
MEXZOG_Hcorrected.cif	MEXZOG_Hcorrected_mi_ucf.cif
[UKIRAI]	UKIRAI_org_mi_ucf.cif
See Li *et al.*, 2014	UKIRAI_corrected_mi_ucf.cif

[a] The energy-minimised structures of the original and the corrected structures are the same.

[b] LAKKEO is an XRPD structure with an incorrectly positioned C atom; LAKKEO01 is an SX structure with incorrectly positioned H atoms.

Space-group revisions

The unit-cell parameters and the positions of the non-H atoms are averages from the original publications, the positions of all H atoms are the result of a DFT-D energy-minimisation with the unit cell and positions of the non-H atoms kept fixed and the new space-group symmetry imposed.

Experiment	Energy-minimisation
[HMBENZ06]	HMBENZ06_org_mi_ucf.cif
HMBENZ06_SpGr_reint.cif	HMBENZ06_SpGr_reint_mi_ucf.cif
[QAMXUY01]	QAMXUY01_org_mi_ucf.cif
QAMXUY01_SpGr_reint.cif	QAMXUY01_SpGr_reint_mi_ucf.cif
[TMAPCL05]	TMAPCL05_org_mi_ucf.cif
TMAPCL05_SpGr_reint.cif	TMAPCL05_SpGr_reint_mi_ucf.cif
[ZZZUXA02]	ZZZUXA02_org_mi_ucf.cif
ZZZUXA02_SpGr_reint.cif	ZZZUXA02_SpGr_reint_mi_ucf.cif
